# Genome composition and GC content influence loci distribution in reduced representation genomic studies

**DOI:** 10.1186/s12864-024-10312-3

**Published:** 2024-04-25

**Authors:** Carles Galià-Camps, Cinta Pegueroles, Xavier Turon, Carlos Carreras, Marta Pascual

**Affiliations:** 1 Departament de Genètica, Microbiologia i Estadística, Universitat de Barcelona, Avinguda Diagonal 643, Barcelona, 08028 Spain; 2 Institut de Recerca de la Biodiversitat (IRBio), Universitat de Barcelona (UB), Barcelona, Spain; 3 Department of Marine Ecology, Centre d’Estudis Avançats de Blanes (CEAB-CSIC), Accés Cala Sant Francesc 14, Blanes, 17300 Spain

**Keywords:** Protostomes, Deuterostomes, Plants, Genome categories, Restriction enzyme, Secondary selection

## Abstract

**Background:**

Genomic architecture is a key evolutionary trait for living organisms. Due to multiple complex adaptive and neutral forces which impose evolutionary pressures on genomes, there is a huge variability of genomic features. However, their variability and the extent to which genomic content determines the distribution of recovered loci in reduced representation sequencing studies is largely unexplored.

**Results:**

Here, by using 80 genome assemblies, we observed that whereas plants primarily increase their genome size by expanding their intergenic regions, animals expand both intergenic and intronic regions, although the expansion patterns differ between deuterostomes and protostomes. Loci mapping in introns, exons, and intergenic categories obtained by in silico digestion using 2b-enzymes are positively correlated with the percentage of these regions in the corresponding genomes, suggesting that loci distribution mostly mirrors genomic architecture of the selected taxon. However, exonic regions showed a significant enrichment of loci in all groups regardless of the used enzyme. Moreover, when using selective adaptors to obtain a secondarily reduced loci dataset, the percentage and distribution of retained loci also varied. Adaptors with G/C terminals recovered a lower percentage of selected loci, with a further enrichment of exonic regions, while adaptors with A/T terminals retained a higher percentage of loci and slightly selected more intronic regions than expected.

**Conclusions:**

Our results highlight how genome composition, genome GC content, RAD enzyme choice and use of base-selective adaptors influence reduced genome representation techniques. This is important to acknowledge in population and conservation genomic studies, as it determines the abundance and distribution of loci.

**Supplementary Information:**

The online version contains supplementary material available at 10.1186/s12864-024-10312-3.

## Background

The availability of genomes is blooming. In the last five years, methodological advances in the sequencing of long fragments have enhanced exponentially the quantity and quality of genomic resources, and several initiatives from global to regional scope have arisen aiming to produce genomes of all biodiversity [1, 2]. Nonetheless, when working with species with big genome sizes or without reference genomes, most genomic studies rely on reduced genome sequencing techniques using restriction site digestion enzymes (RAD) to obtain genome-wide markers of targeted species [3–6]. In population genomics, RAD methods allow working with many individuals without compromising SNP calling accuracy, since high sequencing depth is required for reliable genotyping [7, 8]. The distribution of the sites digested by restriction enzymes is expected to be random, and the resultant fragments are assumed to mirror the genomic structure of the original genome [7, 9]. Consequently, the percentage of loci in a genomic category should be representative of the percentage of the genome in that same category. This would imply a uniform distribution of recognition sites, in which the nucleotide content of the genome nor the nucleotide content of the enzyme’s target have an effect on the distribution of digested fragments. However, whether this assumption holds true across taxa, needs to be properly evaluated, as recent simulated and empirical studies with fish and sea urchins demonstrated that some genomic regions are enriched according to the GC content of the enzyme recognition site [10].

In this context, genome availability provides an unprecedented opportunity to dig deep into the genome composition of living organisms [11, 12]. Recent studies have shed light on plant genome sizes [13], abundance of repeated elements in mammals and arthropods [14, 15], and the existence of duplicated regions in insects and hexapods [16, 17]. Also, the genome GC content has been explored in detail [18, 19], and the percentage of intergenic and genic regions has been evaluated in both plants and animals [20, 21]. Genome evolution processes are complex and involve many heterogeneous mechanisms among taxa. All these processes might impact population genomic studies, as a key element when assessing genomic structural variants and during SNP calling [22]. However, genomes across different taxonomic groups should be analyzed using the same methodology for a correct inference of the features that might be relevant for population genomic studies using RADseq techniques.

Among RAD techniques, 2b-RAD allows working with degraded DNA [23]. This is because it uses 2b-enzymes that identify a recognition site of 6–8 bp and cleave DNA upstream and downstream at a given length, generating small fragments of 32–34 bp with sticky ends including a few random nucleotides [24]. All generated fragments can be sequenced following standard protocols with the ligation of fully degenerated adaptors to the sticky ends for library building. Fully degenerated 2b-RAD adaptors are characterized by two terminal “N”s, which allow binding to all fragments resulting from digestion. Nonetheless, this enzyme family allows the use of base-selective adaptors, which select fragments with specific nucleotides in their sticky ends [8, 9, 23]. This unique feature of 2b-RAD technique provides the option to perform a secondary reduction of the number of loci, allowing to work with species with large genomes at a reduced cost, and therefore including many more individuals given a locked budget. Compared to the first reduction, produced by the enzymes’ recognition sites, secondary reduction via base selection could be directed to specific regions rich in nucleotides fixed in the base selective adaptors. However, this assumption has not been formally tested, and the number and distribution of loci retained after base selection needs to be evaluated.

Here, we aim to analyze the genomic composition between taxonomic groups, and to assess its effect on the number and distribution of loci obtained with reduced representation sequencing techniques using different enzymes and base selective adaptors. To do so, we downloaded 80 publicly available reference genomes from three major taxonomic groups (plants, protostomes, and deuterostomes) and evaluated the relative abundance of three genomic categories (intergenic, intronic, and exonic). We simulated the digestion of three 2b-enzymes on the downloaded genomes to test the number and distribution of the loci potentially being recovered in population genomic studies, according to genome size and taxonomic group. Finally, we simulated the effect of two types of base selective adaptors, with different GC content, to determine the number and distribution of loci obtained in the three genomic categories with this secondary reduction. Our results show how genome architecture influences reduced genome representation techniques in terms of number and distribution of loci, which must be acknowledged in population and conservation genomics.

## Materials and methods

### Reference genome datasets

**Fig. 1:**
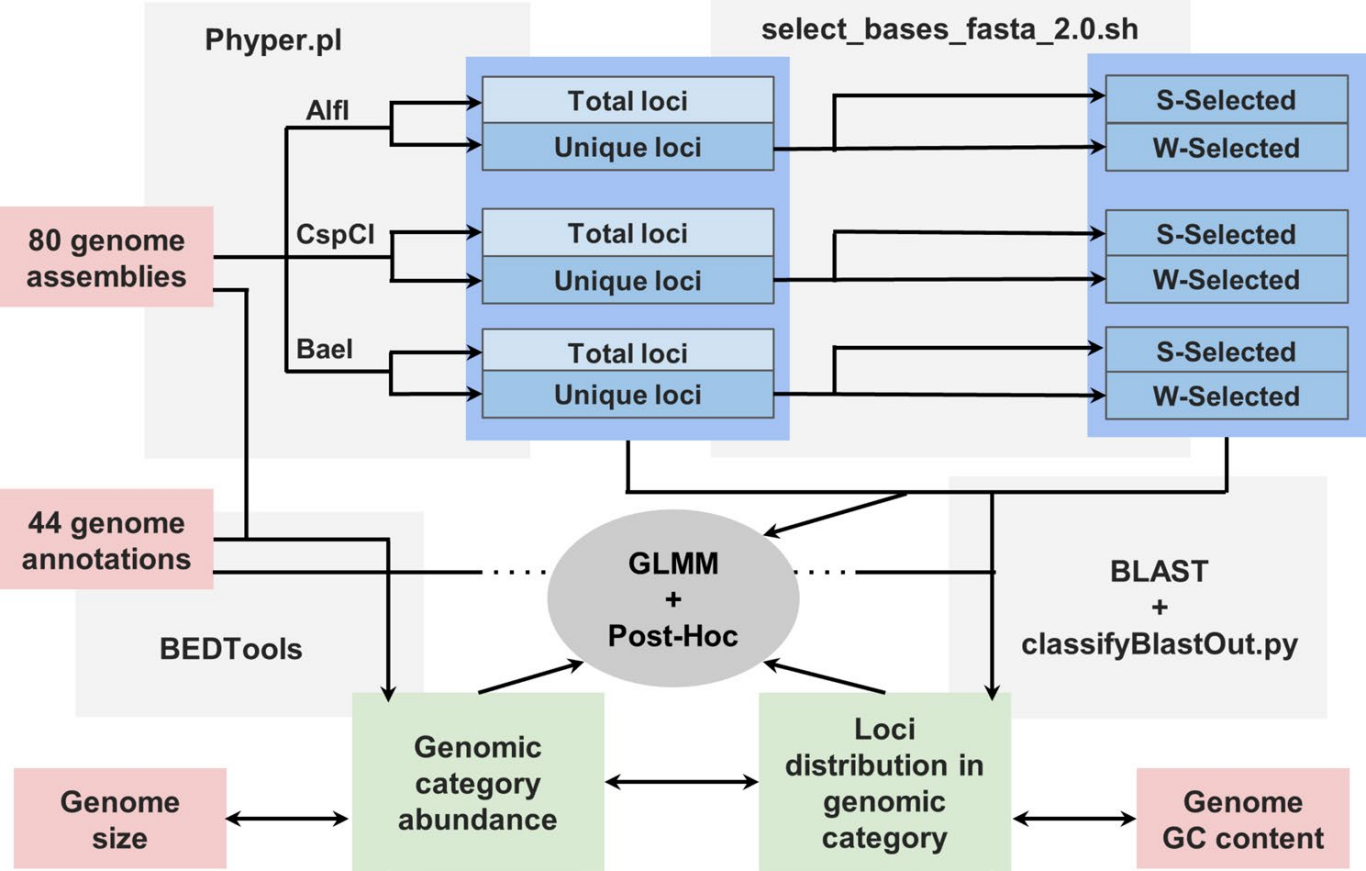
Schematic flowchart followed in the present study. Red boxes identify initial datasets obtained from public databases, blue boxes identify intermediate datasets, green boxes identify final datasets, and gray boxes identify steps where a software was applied. Note that for “Total loci”, in light blue, only GLMM and Post-Hoc were carried out. Single headed arrows indicate the flow of the processes, while double headed arrows indicate that correlations were carried out between two datasets

**Fig. 2:**
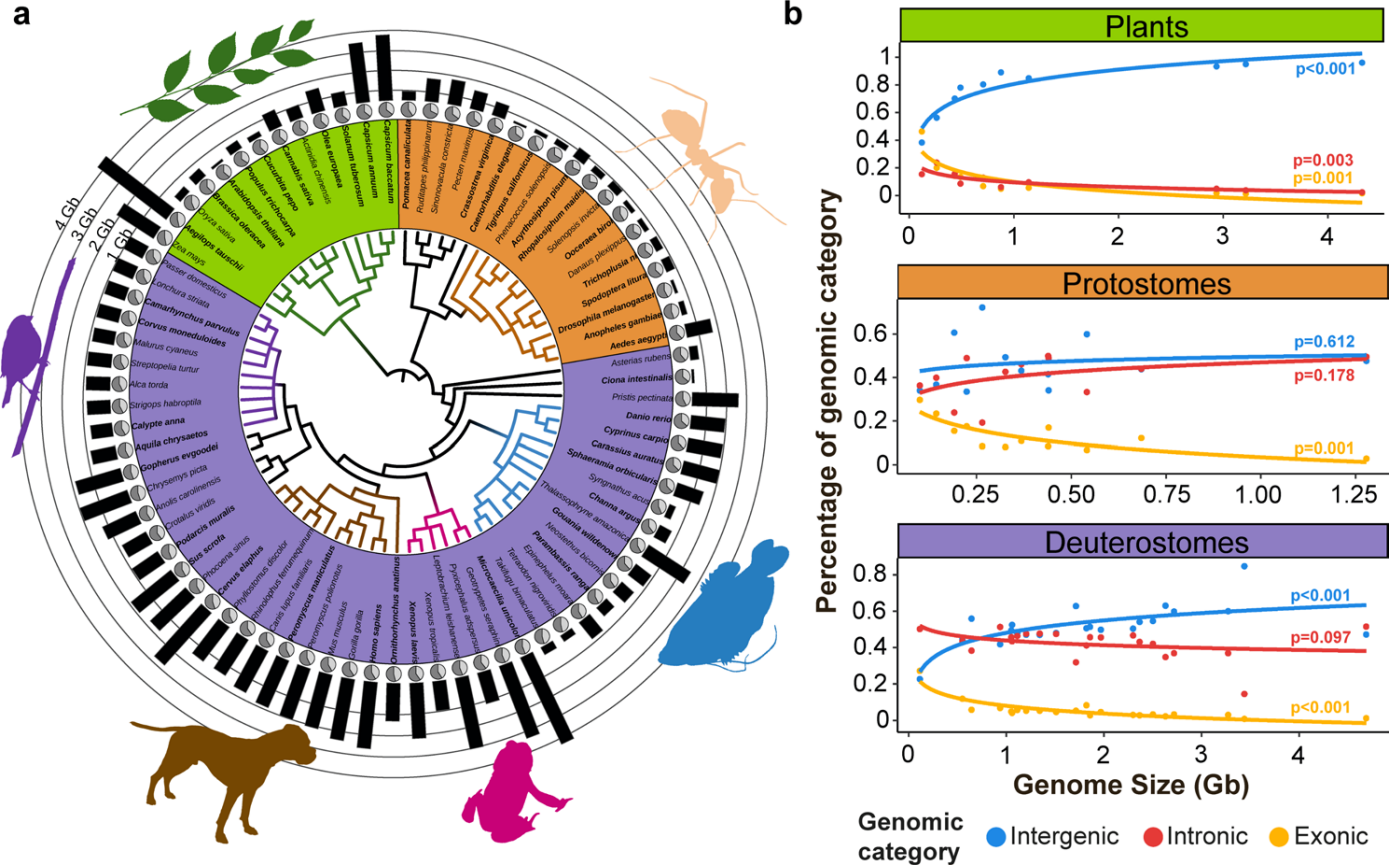
Phylogenetic representation of the 80 genomes used and their genomic composition. (**a**) Phylogenetic tree, in which monophyletic groups with six or more species are indicated by colored branches and identified with a shape (Plants = green, Arthropods = orange, Fishes = blue, Amphibians = pink, Mammals = brown and Birds = violet). The species names are highlighted with background color according to the three Supergroups considered (Plants = green, Protostomes = orange, Deuterostomes = purple). Species names in bold indicate genomes with annotation information. Pie diagrams indicate the GC content of each species (GC = Light gray, AT = Dark gray), and bars their genome sizes. (**b**) Percentage of intergenic, intronic and exonic regions related to genome size for the 44 species with annotated genomes grouped by supergroup (Data S1)

We downloaded and analyzed 80 reference genome assemblies publicly available from GeneBank (Fig. 1). They ranged from 102 Mb to 4.7Gb, and comprised both plants and animals (Data S1). Information on the number of contigs, L50, N50, and GC content for each genome (Data S1) was obtained with ABySS-2.4.4 [25]. A benchmarking of universal single copy orthologs (BUSCO) was conducted for all genome assemblies using the software BUSCO-5.3.2 and the dataset eukaryota_odb10 [26] to ensure that the reference genomes are of decent quality. The percentage of complete BUSCO values ranged from 89.8 to 100% with a mean value of 97.11 ± 2.67 (Data S1). We obtained a phylogenetic tree of the selected taxa using Timetree web server (http://www.timetree.org/) [27]. We defined 3 different supergroup clusters, composed of 13 plants, 18 protostomes, and 49 deuterostomes (Data S1) (Fig. 2a). Additionally, a total of 12 different groups were defined: plants (13), molluscs (5), nematodes (1), arthropods (12), echinoderms (1), tunicates (1), fishes (14), amphibians (6), mammals (12), lepidosaurs (3), testudines (2) and birds (10). The groups with more than six species were further evaluated separately (Fig. 2a). We retrieved the annotation files of the same genomes in GFF format, obtaining the annotation for 44 genomes (Fig. 1): 10 plants, 2 mollusks, 1 nematode, 9 arthropods, 1 tunicate, 7 fishes, 2 amphibians, 5 mammals, 1 lepidosaur, 2 testudines, and 4 birds (Fig. 2a, Data S1).

### Genomic composition and functional categories

For every annotated genome assembly, we calculated the number of base pairs in exons, introns and intergenic regions, herein designated as genomic categories, using the genomecov function with -d -split options from BEDTools (Fig. 1, Data S1) [28]. To do so, we first converted the GFF files to bed12 format using the gff3_file_to_bed.pl utility from Transdecoder (Haas, BJ.https://github.com/TransDecoder/TransDecoder).

### Genomic in silico digestions

We computationally digested the 80 genomes with 2b-enzymes using the program Phyper.pl [29] (Fig. 1). This program recognizes 2b-enzyme targets, cleaves the DNA, and exports the obtained fragments. We carried out the analyses with three 2b-enzymes with different GC content in their recognition sites: AlfI ([10/12]GCA[N6]TGC[12/10], 66% GC), CspCI ([11/13]CAA[N5]GTGG[12/10], 57% GC) and BaeI ([10/15]AC[N4]GTAYC[12/7], 50% GC). For every in silico digestion, we obtained two fasta files corresponding to the total (all digested fragments) and unique (fragments that were present only once in the genome) sequences [29] (Fig. 1). Unique loci are of prime interest in population genomic analysis, since loci found in multiple locations are routinely removed in the filtering steps. To reduce the effect of genome size, as larger genomes are expected to provide more fragments than smaller genomes, we calculated the percentage of unique loci to standardize the data for comparisons.

### In silico digestions simulating the use of base-selective adaptors for secondary reduction

We simulated the effect of using base-selective adaptors with the bash script select_bases_fasta_2.0.sh [23] (Fig. 1). We performed in silico base selections of the unique loci ending with G or C (S) sticky ends (terminal nucleotides are G-G, G-C, C-C, and C-G) and ending with A or T (W) sticky ends (terminal nucleotides are A-A, A-T, T-T, and T-A) in order to determine the percentage of unique loci that would be retained with these secondary selections, since they can be easily implemented in empirical and in silico studies [8, 23]. Finally, we calculated the percentage of retained sequences after the selection with S- and W-adaptors compared with the initial number of unique loci for each of the 80 species and each of the three enzymes.

### Categorical profiling of 2b-RAD loci

To calculate which proportion of unique loci correspond to intergenic, intronic, and exonic categories, we first selected the sequences of the unique loci resulting from the in silico digestion with the three enzymes for the 44 annotated genomes. To identify the location of unique loci in their corresponding reference genome we used BLAST [30] (same size, 100% of identity, e-value = 1^10^− 16^) (Fig. 1). Afterward, we used the in-house script classifyBlastOut.py pipeline to classify unique hits as exonic, intronic, or intergenic (https://github.com/EvolutionaryGenetics-UB-CEAB/classifyBlastOut/)(Fig. 1). The blast hits that included both exonic and intronic regions were classified as exonic. Finally, we estimated the percentage of unique loci corresponding to each genomic category in S-selected and W-selected datasets for each annotated genome and enzyme.

### Graphics and statistical analyses

Dispersion plots and violin plots were drawn with the R package “ggplot2” [31] Regression formulas and their R^2^ and p-values were calculated using the “stats” package from R (Fig. 1). General Linear Mixed-Effects Models (GLMMs) were estimated with the R package “lme4” [32], and “car” [33] was used to assess statistically significant effects of the explanatory factors (Fig. 1). Normalization of the data was achieved through an arcsine-square root transformation for factors with frequency values (i.e.: percentage of unique loci, percentage of genome in each genomic category and percentage of unique loci in each genomic category). Normalization of the data was achieved through a logarithmic transformation for factors with count values (i.e.: number of total loci, number of unique loci, and genome size). The package “rsq” [34] was used to check the coefficient of determination (R^2^) for the whole model and for the fixed factors included. For statistically significant effects, multiple-level post-hoc comparisons were carried out with the function *contrast* of the R package “emmeans” [35] applying a Tukey-adjustment of the p-value to avoid Type I errors (Fig. 1), and plots were generated with the function *emmip* from the same package.

## Results

### Evolutionary trends on genome composition

The compilation of the 80 genomes (Fig. 2a, Data S1) highlighted different trends among supergroups (plants, protostomes, and deuterostomes) regarding how the three considered genomic categories (intergenic, intronic, and exonic) change in relation to genome size (Fig. 2b). In all three taxonomic supergroups, species with small genome sizes had a higher percentage of exonic regions, as shown by the significant negative slopes of their regression equations and their high coefficient of determination (Fig. 2b, Table S1). The percentage of intergenic regions in plants increased with genome size, while both intronic and exonic regions were found at low percentages and with significant negative regressions with genome size (Fig. 2b, Table S1). On the other hand, animal genomes increased in size by expanding both intergenic and intronic regions (Fig. 2b). However, the two animal supergroups differed in the increment pattern of these categories with genome size (Fig. 2b). Protostomes increased genome size by proportionally increasing intergenic and intronic regions, which are found at high percentages in their genomes. Deuterostomes also showed a high percentage of intergenic and intronic regions, but when increasing genome size only intergenic regions increased significantly (Table S1). In accordance with these observations, General Linear Mixed-Effects Models (GLMM) using the percentage of each genomic category as the dependent variable detected significant differences in the interactions considering the three factors: Genomic category, Supergroup, and Genome size (Table S2). For the significant interaction between Genomic category and Supergroup, no differences in exonic regions were observed by the post-hoc tests between taxonomic groups. However, the percentages of intronic and intergenic regions were significantly different between plants and animals (Table S3).

### In silico genome digestions using 2b-RAD enzymes

**Fig. 3:**
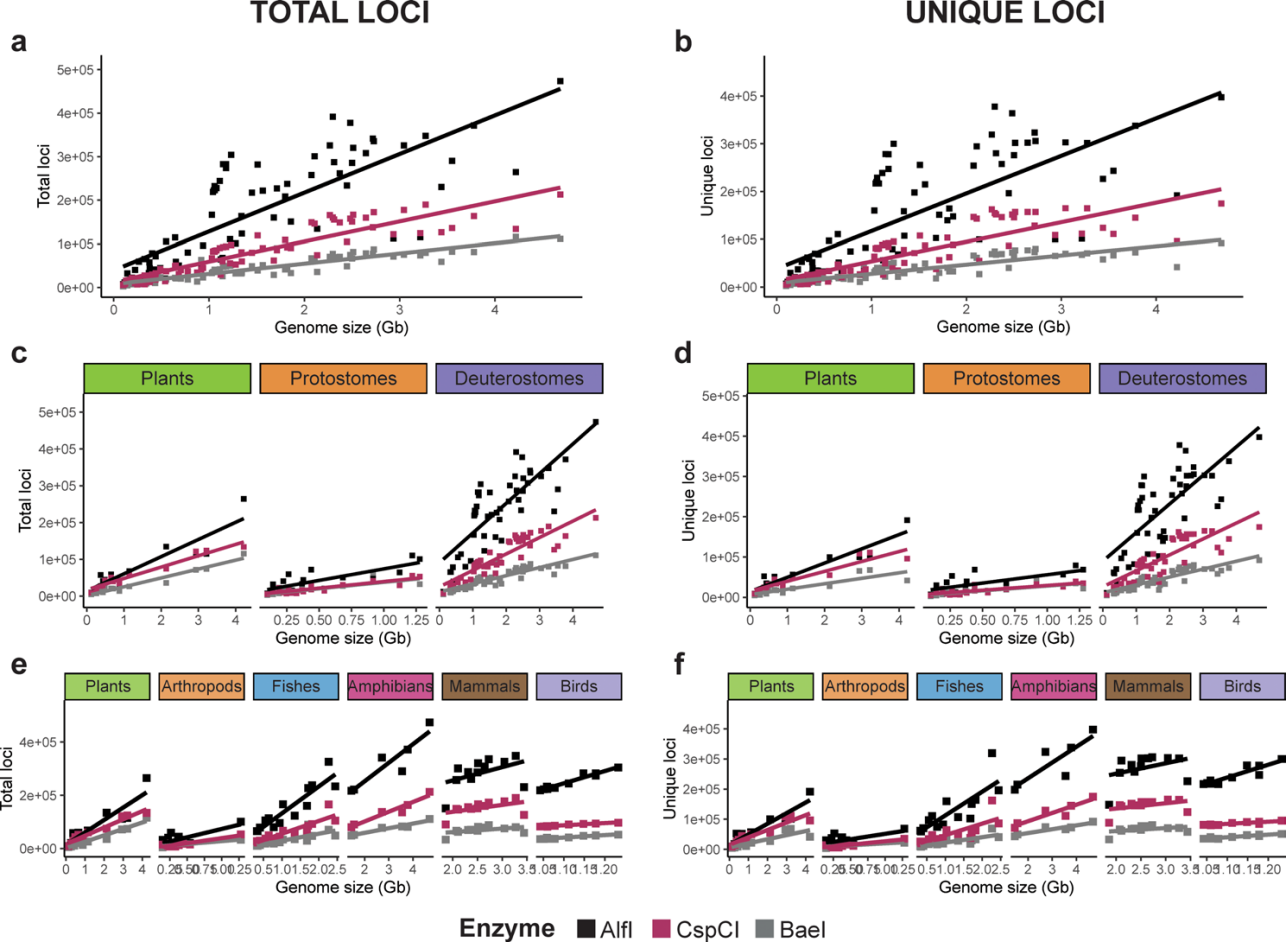
Linear regressions of the loci yielded by each enzyme (AlfI, CspCI, BaeI) according to each species’ genome size for total and unique loci. (**a**-**b**) Linear regressions considering all 80 genomes. (**c**-**d**) Linear regressions for each supergroup independently (plants, protostomes and deuterostomes). (**e-f**) Independent linear regressions for those groups with six or more species (plants, arthropods, fishes, amphibians, mammals and birds). Regression equations can be found in Table S4

Our results from the in silico digestion of the 80 genomes with the three 2b-enzymes, showed that the number of total loci (all fragments) and unique loci (those fragments whose sequence was present only once in the genome) were highly correlated (Figure S1). The number of total and unique loci significantly increased with genome size in all enzymes, regardless of taxonomic level, presenting high coefficients of determination (R^2^) for most of the regressions (Fig. 3, Table S4). Mammals were the exception to the global trends (Table S4), since two genomes in this group (platypus and red deer, the smallest and largest genomes analyzed, respectively) had low numbers of loci (Data S1).

In the three GLMM models tested (Table S5), considering all species together (Total model), split by supergroup (Supergroup model) or by group considering those with 6 or more species (Group model), the proportion of the variance explained by the fixed factors Enzyme, Genome size, and Supergroup or Group (depending on the model), was high. For the Total model, significant differences were found among enzymes (AlfI, CspCI, BaeI), with genome sizes, and their interaction (Table S5). Differences were due to the higher number of loci obtained with AlfI, being this number intermediate for CspCI and smallest for BaeI, and to the increase of the number of loci with genome size, with different slopes for each enzyme (Fig. 3, Table S4). For the Supergroup model, the fixed factors explained 92% of the variance for total loci and 90% for unique loci. Supergroup, enzyme and genome size had significant effects, as well as the interaction enzyme*supergroup in both total and unique loci (Table S5). Tukey’s post-hoc pairwise comparisons indicated major significant differences between deuterostomes and the other two taxa for all enzymes but BaeI (Table S6). Similar results were obtained when considering the group model, with 93% of the variance explained by the fixed factors (Table S5). As in the Supergroup model, no differences between taxonomic groups were found when using BaeI (Table S7). However, AlfI presented significant differences in total and unique loci when comparing plants and arthropods against the other groups as assessed with Tukey’s post-hoc tests (Table S7). For CspCI, significant differences were only found when comparing arthropods’ unique loci with other groups.

### The percentage of unique loci varies across taxa

**Fig. 4:**
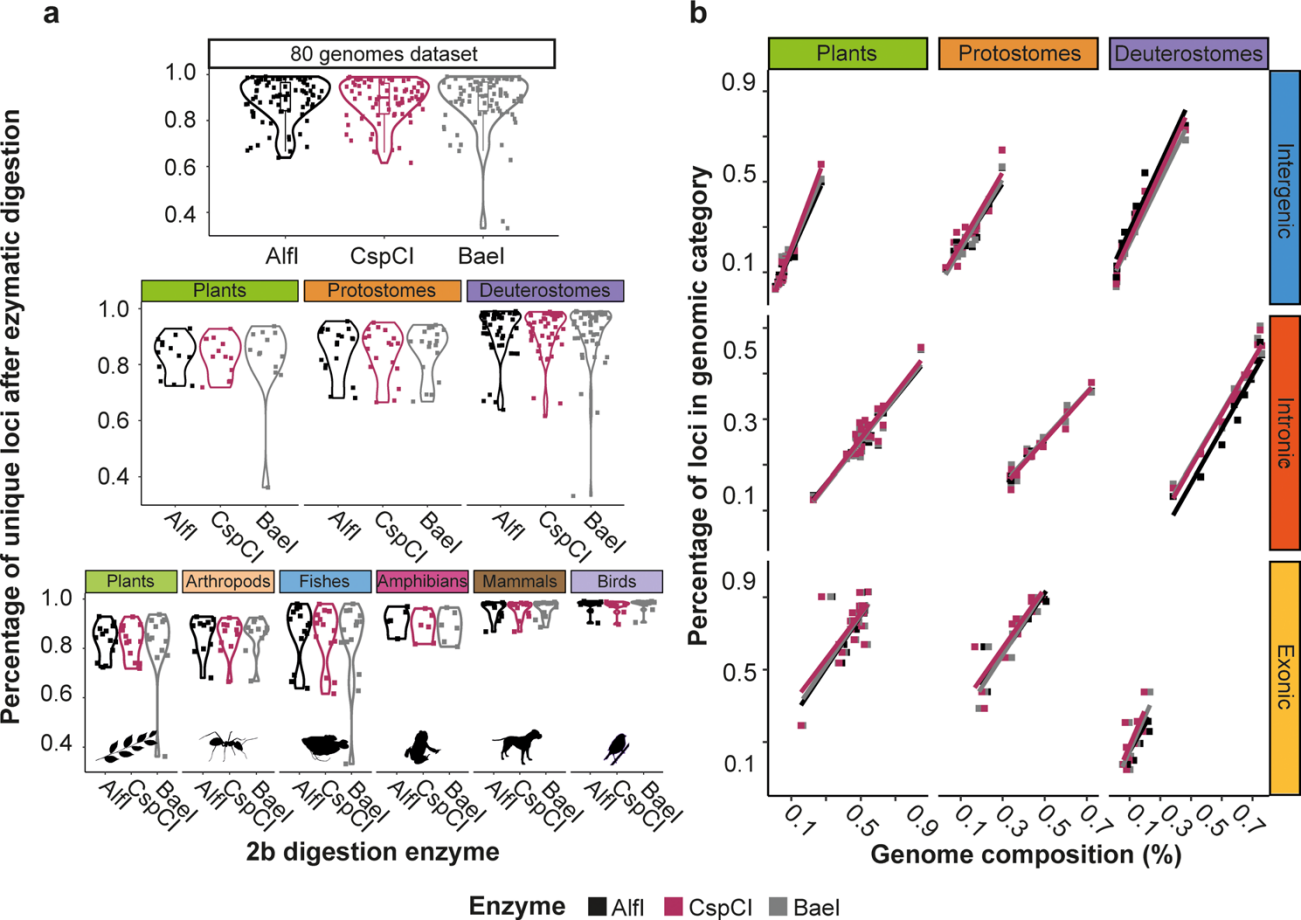
Percentage of unique loci and genomic category distribution across taxa. (**a**) Violin plots of the percentage of unique loci, in relation to the total number of loci, obtained after in silico genome digestion with 2b-enzymes. (**b**) Percentage of unique loci assigned to each genomic category (intergenic, intronic and exonic) compared to the percentage of the same genomic category in the genome. Dotted lines indicate the percentage of loci in a genomic category expected under the null hypothesis of random distribution of loci

Our GLMM showed that the percentage of recovered unique loci relative to the number of total loci was not different across enzymes and did not change with genome size when considering all species together (Fig. 3a, Table S8). However, these percentages were dependent on the taxa analyzed, since the variance of the full model explained by fixed factors increased when the species were combined in lower-level phylogenetic groups, suggesting lineage-specific variation (Table S8). The Supergroup model showed significantly higher percentages of unique loci on deuterostomes than on plants and protostomes (Fig. 4a, Table S9). Finally, the Group model showed different behaviors depending on the groups, since mammals (0.958 ± 0.036, mean ± SE) and birds (0.972 ± 0.027) presented a higher percentage of unique loci (Fig. 4a). However, this effect was only significant in mammals when compared to plants, arthropods, or fishes (Table S10). Birds did not show significantly different values despite their high percentage of unique loci and low dispersion values (Fig. 4a). The model in birds presented a large 95% confidence interval on the percentage of unique loci, which overlapped with all other groups (Figure S2).

### 2b-RAD digestions slightly enrich exonic loci

We BLASTed unique loci to their reference annotated genomes and identified to which genomic category they belonged (Data S2). For all enzymes and species being tested, all loci BLASTed randomly over the entire reference genomes indicating that loci were not aggregated, thus mirroring reference genomes without biases towards any specific region. For the three supergroups, the percentage of unique loci in a given category significantly increased with the percentage of the same genomic category in the genome (Fig. 4b, Table S11, Data S2). Overall, the percentage of variation explained by all regression equations was very good, as indicated by the coefficients of determination of the full models (R^2^, Table S11). Loci mapping in exonic regions were more frequent than expected, since the values fell above the percentage of unique loci in a given genomic category expected under the null hypothesis of random distribution of loci (dotted line y = 0 + 1x, Fig. 4b). On the contrary, the percentage of loci in intergenic regions were inferior to the expected ones as they always fell below the dotted line (Fig. 4b). Finally, the intronic regions had a percentage of unique loci roughly proportional to the percentage of the corresponding category (close to the dotted line, Fig. 4b).

The GLMM using as the dependent variable the ratio between the percentage of unique loci in a genomic category and the percentage of the same category in the genome, identified significant differences among enzymes, supergroups, genomic categories, and their pairwise interactions (Table S12). For the pairwise interactions, there were significant differences between genomic categories for all enzymes with the exception of the comparison between intergenic and intronic categories for AlfI and BaeI (Table S13). The percentage of unique loci in intergenic regions did not differ between supergroups. However, plants had a significantly higher proportion of unique loci in the exonic category than animals, and protostomes had a significantly higher proportion of unique loci in the intronic category than plants. All genomic categories had significantly different proportions of loci in each supergroup except the comparisons between intergenic and intronic regions for plants and deuterostomes (Table S13, Figure S3).

### Base selection performance depends on genome GC content

**Fig. 5:**
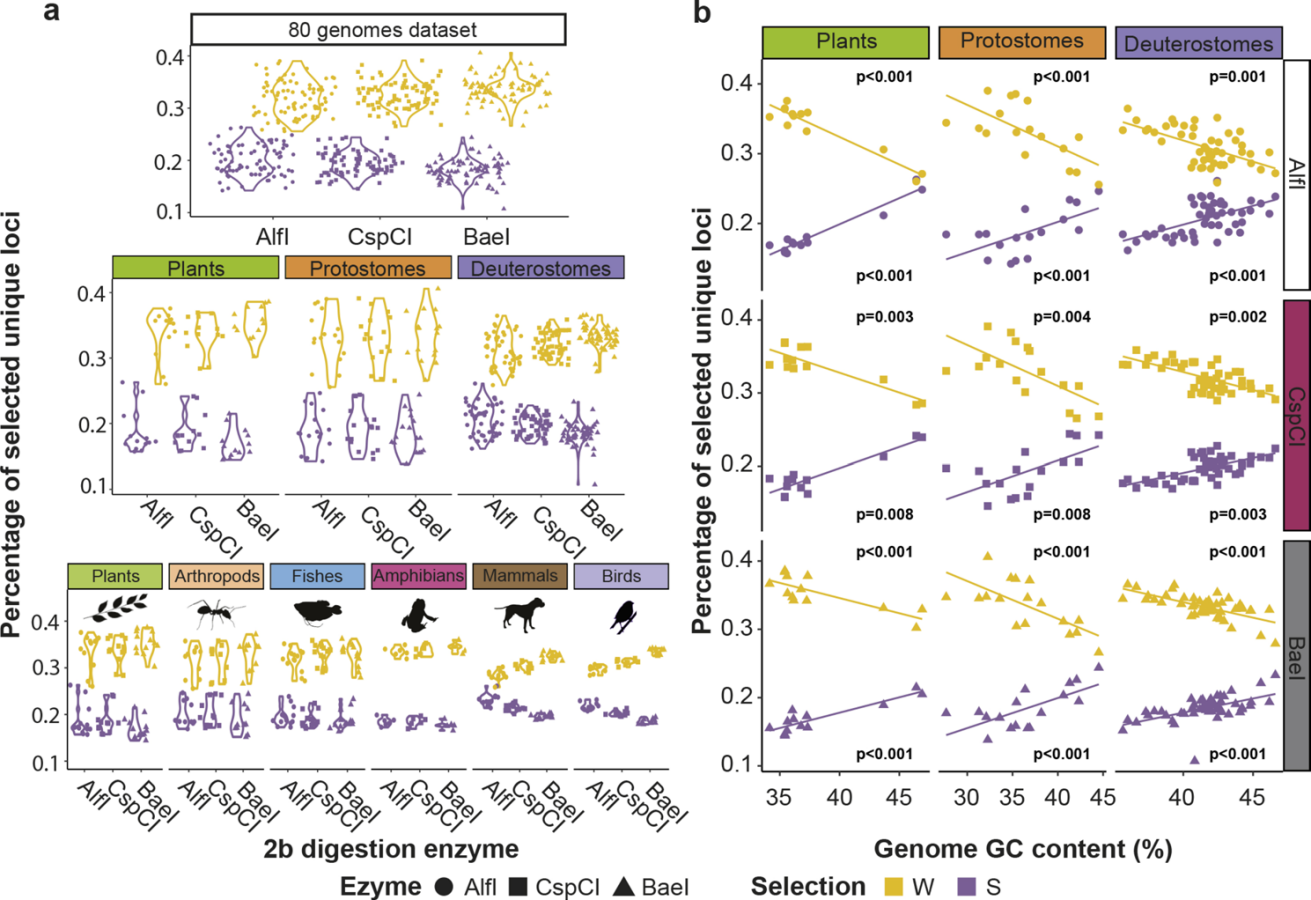
Percentage of retained unique loci across taxa when using base selective adaptors. (**a**) Percentage of unique loci retrieved when S (GC) and W (AT) base-selective adaptors are in silico simulated for each enzyme (AlfI, CspCI, BaeI). (**b**) Linear regressions of the percentage of selected loci retained according to the genome’s GC content

Our results simulating the use of base-selective adaptors on 80 genomes showed a great reduction of the number of loci highly dependent on whether the selection was performed for S or W sticky ends, providing ∼ 0.19% and ∼ 0.33% of original unique loci respectively compared to not using base selection (Fig. 5). The percentage of unique loci retained with W-selection was significantly higher than for S-selection (Table S14, Fig. 5a). Additionally, significant differences were found for some of the interactions when simulating adaptor selection for the Total, Supergroup and Group models (Table S14). For the Total model, BaeI presented a significantly lower percentage of retained unique loci for both S and W-selection when compared to AlfI, and only for S-selection compared to CspCI (Table S15). For the Supergroup model, a significant interaction was found between selection and supergroup. Deuterostomes retained a significantly higher percentage of unique loci with S-selection and lower with W-selection than the other two supergroups (Table S16, Fig. 4a). For the Group model, mammals showed significantly higher percentage of unique loci retained with S-selection and lower with W-selection when compared to all other groups but birds (Fig. 5a, Table S17). For all groups but birds there was a significantly higher percentage of loci retained with W-selection (Table S17, Figure S4).

The number of retained loci could be highly influenced by the nucleotide content of the genomes, generally richer in AT than in GC (Data S1). We observed that the percentage of unique loci retained with S-selection within each supergroup was positively correlated with the species genome GC content, while the percentage of loci retained with W-selection was negatively correlated with the species GC content (Table S18, Fig. 5b). Thus, more loci were retained with S-selection when the genome GC content was higher, and the reverse occurred with W-selection (Fig. 5b). Overall, considering the species GC content, which is on average 39.93 ± 3.9% (Data S1), the expected mean number of loci retained with the S-selection for the 80 analyzed species would be 15.94% (% of GC^2^/100). Similarly, the W-selection expected number of loci given an average AT content of 60.07 ± 3.9% would be 36.08% after applying the same equation (% of AT^2^/100). The observed values of percentages of loci retained with W-selection (32.7 ± 0.029%) and S-selection (19.2 ± 0.025%) in the simulations with the 80 genomes were quite similar to those expected considering only the genomes’ GC content.

### Base selection enhances differential recovery of genomic categories

**Fig. 6:**
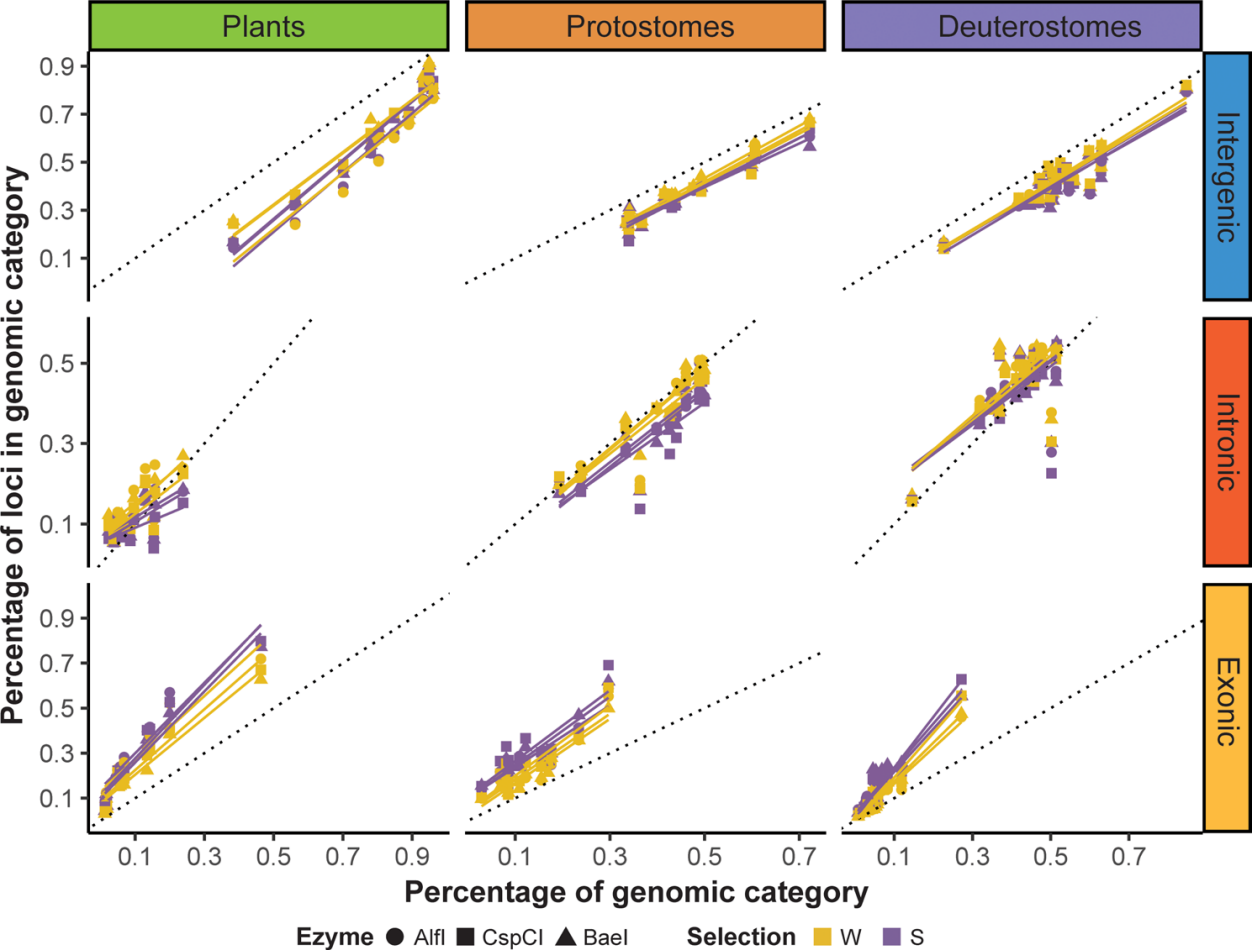
Percentage of base-selective unique loci assigned to each genomic category compared to the percentage of the same genomic category in the genome. The dotted lines indicate the percentage of loci in a genomic category expected under the null hypothesis of random distribution of loci

After base-selection, we BLASTed the selected unique loci to their reference annotated genomes and identified to which genomic category they belonged (Data S2). As found for unique loci, all selected loci were randomly distributed across their respective reference genome. The percentage of unique loci in a genomic category was again highly correlated with the percentage of the same category in the genome (Table S19). As found without base selection, the percentage of unique loci was above the expectation (dotted line, Fig. 6) for exons, below for the intergenic regions, and the closest to the expectation for introns. However, base selection also had an effect on top of this general pattern, as the regression lines obtained for all enzymes using the S-selection were always above those obtained with the W-selection in exons. This pattern was reversed for the intronic regions, in which W-selection regression lines were above those of the S-selection ones.

The General Linear Mixed-Effects Models (GLMM) showed that all pairwise interactions were significant except for the enzyme*selection interaction (Table S20). The triple interaction involving enzyme, supergroup and genomic category was also significant (Table S20). All the post-hoc tests for the selection and genomic category interaction resulted in significant values with the exception of the comparison between S- and W-selection in the intergenic regions (Table S21). The selection type directly affected the percentage of unique loci in a given genomic category with a significantly higher percentage of intronic loci in W-selection and a significantly higher for exonic regions in S-selection (Table S21). Regarding the interaction between supergroup and selection, only deuterostomes showed significant differences between S- and W-selection with a higher proportion of unique loci recovered by the former (Table S21). When comparing the different supergroups, plants presented a higher proportion of unique loci with W-selection than protostomes and deuterostomes (Table S21). The three-way interaction showed that all supergroups presented significant differences in the dependent variable between genomic categories in the same direction regardless of the used enzyme, with the exception of protostomes, which did not show significant differences between intergenic and intronic regions with any of the three enzymes (Table S22). Plants showed a significantly higher ratio of unique loci in exons compared to animals with all three enzymes (Table S22). Protostomes showed significantly lower values in introns than plants when using the enzymes AlfI and BaeI. Finally, there were no significant differences between enzymes with the exception of AlfI recovering more loci in the exons of plants (Table S22).

## Discussion

In agreement with previous studies, our results show that plants and animals increase genome size by differently expanding intergenic and intronic genomic categories respectively [20, 21]. We confirm that the genome size and composition, in combination with the GC content of the restriction enzyme target site, determine the number and distribution of loci in studies based on reduced representation sequencing techniques. Moreover, we prove that the number of markers in the different genomic categories with 2b-enzymes positively correlates with the percentage of each region in the genome, although with a slight enrichment of loci in exons. Finally, our simulations of 2b-RAD secondary reduction techniques show how each genome category GC content influences the number and distribution of retained loci as a function of the base-selective adaptors used.

### Genomic composition is shaped by multiscale evolutionary processes

Our results indicate that the genome composition differs among supergroups, which might determine the loci distribution in reduced representation population genomic studies. In the case of plants, we have shown that they expand their intergenic regions with genome size. Allopolyploidy has been a main evolutionary trigger for plants, since 87.5–99.5% of plants have been subjected to hybridization at some point during their evolutionary history, with a posterior rediploidization [36]. This process involves many genomic changes, with fast gene deletion being one of the predominant mechanisms [37]. Retained homeologs in plants enhance protein family diversity without relying on introns to create different isoforms through alternative splicing, resulting in a higher number of genes [36, 38, 39]. Thus, allopolyploidy may help to maintain the exonic and intronic regions at low proportion despite increasing genome sizes, as observed in our study. Furthermore, the high percentage of intergenic regions in plants compared to animals is in agreement with plants increasing their genomes by expansions of transposable elements (TE) that can be activated by hybridization and polyploidization altering silencing mechanisms [40, 41]. The complexity of plant genomes deserves further detailed studies with more species of differing architectures.

Conversely, we found that intronic regions were highly abundant in animals across genome sizes. The abundance of intronic regions has been proposed as a mechanism to facilitate alternative splicing, contributing to the diversification of gene structural variants in animals [42]. Furthermore, we also found that in deuterostomes the percentage of intergenic regions significantly increases with genome size. In the origin of vertebrates, two ancient rounds of whole genome duplications 450 Mya occurred [43]. These duplication events followed by TE activity might have increased the proportion of intergenic regions, since the effect of TE could inactivate former duplicated genes and thus contribute to the expansion of intergenic regions at the expense of ancient genes [44, 45]. Furthermore, regulatory elements modulating gene expression, highly abundant in vertebrates, have been identified in intergenic regions [46, 47]. However, the information on regulatory elements is limited to a few species due to the lack of comprehensive annotations in most organisms, highlighting the need for correct annotation for the increasing number of available reference genomes.

### 2b-RAD sequencing mostly mirrors genome composition

The absence of biases in the number and distribution of loci being retained by reduced genome representations on population genomic studies is a prerequisite for these studies that has been assumed without evidence. By using three different 2b-enzymes in three major eukaryotic lineages, we have found that, as expected, the number of total and unique loci increased overall with genome size in all enzymes regardless of taxonomic level. The number of unique loci is a key parameter for RADseq studies, as they are the only ones to pass the filtering processes. Although their number positively increased with genome size, not all taxonomic groups behaved similarly when considering the percentage of unique loci in relation to the total number of loci in the genome. In this scenario, plants and protostomes showed lower percentage of unique loci in comparison to deuterostomes. The abundance of recent TE expansions in protostomes (increasing the abundance of repetitive regions in the genome), and recent polyploidy and whole genome duplication in plants (due to autopolyploidy or hybridization), might explain the lower proportion of unique loci found in these two groups [15, 16, 38, 48, 49]. Among deuterostomes, mammals and birds share many genomic evolutionary traits, such as an active expansion of TEs and large DNA deletions [44, 50] that might determine [15, 16, 38, 48, 49] the higher percentage of unique loci found in these two groups in the present study. However, birds did not show significant differences compared to the other groups despite their high percentage of unique loci and low dispersion values. Birds are known for having compact genome sizes (ranging from 0.9 to 1.6Gb), thought to be driven by flight constraints [44, 50]. Consequently, the model needs to generate estimates for a large range of values resulting in high confidence intervals. Finally, fishes present a high dispersion in the number of unique loci among species, mostly the result of cyprinids having extremely low values. Cyprinids suffered two extra genome duplications which may explain the lower number of unique loci found in fishes [51, 52]. Overall, we can confirm that the loci obtained from digestion with 2b-enzymes generally reflect genome composition.

Moreover, we demonstrated that the markers obtained with this technique mirror the overall genome category composition, albeit with a slight enrichment of loci from the exonic regions and a reduction of those from the intergenic category. Other RADseq population studies have recently shown that loci in different genomic categories might be enriched depending on the used enzyme, and suggested an effect of the GC content of the recognition site [10]. Our results point in the same direction since the enzymes used in our study (AlfI, CspCI and BaeI) have a percentage of GC content over 50% in their recognition sites. Thus, finding an overrepresentation of loci from exonic regions is expected given that these regions are GC enriched across eukaryotes while introns and intergenic regions overall contain lower GC content levels [19, 53]. Our results support the idea that the GC content of the enzymes’ recognition site influences the genomic categories being recovered in RADseq genomic studies regardless of taxonomic group and genome size. Thus, the effect of different enzymes preferentially targeting certain genomic regions, specifically exons, should be considered in the design of population genomic studies.

### Base-selection unravels additional GC biases

RADseq population studies of species with big genome sizes will require large sequencing effort for a correct genotyping since the number of loci increases with genome size. To reduce the number of loci, enzymes with infrequent target sites or allowing secondary reduction can be used. 2b-RADseq is the only technique that allows a secondary reduction by using base-selective adaptors to further reduce sequencing costs [23]. As expected, we have shown that base-selective adaptors efficiently reduce the number of recovered loci and the percentage of retained unique loci in a genomic category is highly correlated with the percentage of the same category in the genome. Nevertheless, the selection performed on the unique loci and the genome’s GC content influence the number and genomic category of the loci retained by each methodology.

As 2b-RAD secondary reduction by using base-selective adaptors is performed after the digestion process, the recovered fragments are not a reduced representation of the whole genome, but only of the fragments already digested by the enzyme. Accordingly, AlfI, the enzyme with the richest GC content in the recognition site recovers a significantly higher percentage of S-selected loci and lower of W-selected loci than BaeI when considering all species, likely because the first genome reduction already selected a higher proportion of fragments with higher GC content, which are in turn a better target for the S-selection. However, differences are also observed among taxonomic groups in the abundance of retained loci with adaptor selection. Deuterostomes, and more specifically mammals, retain a significantly higher percentage of unique loci with S-selection and lower for W-selection compared to the other taxonomic categories. Finally, we have found that the higher the GC content of the genome, the higher the percentage of loci recovered using S-selection adaptors and the lower the percentage recovered with W-selective adaptors. Thus, we have shown that the number of the retained loci is influenced by the type of selection performed, the taxonomic category, and the genome’s GC content.

Moreover, although loci in exons were already enriched by the initial digestion, the secondary reduction showed that S-selection further enhances the recovery of loci from exonic regions compared to W-selection. These results are in line with the above-mentioned enrichment of GC in exonic regions across eukaryotes, rendering them a better target for S-selective adaptors. On the other hand, the percentage of loci recovered in introns and intergenic regions, albeit overall below expectation after the initial digestion, were higher when using W-selection than S-selection, again reflecting that non-coding regions have poorer GC content. Thus, the percentage of GC in a genomic category can be used as a prior to predict the performance of both S-selective and W-selective secondary reductions.

## Conclusions

Our results highlight different modes of evolution of genomes across broad taxonomic categories. Whereas plants primarily increase their genome size by expanding their intergenic regions, animals expand both intergenic and intronic regions, although the patterns differ between deuterostomes and protostomes. The results presented in this work are also highly relevant to the design of population genomic studies. We have shown that the GC content of the enzymes determines an enrichment of loci in particular genomic regions. Further, the use of base-selective instead of fully-degenerated adaptors provides an efficient means to reduce the number of loci to make the research more cost-effective, but the type of selection reinforces bias in certain genomic categories. Overall, we highlight that the enzyme we used for digestion, type of secondary selection, species’ genomic composition and GC content may determine differences in the distribution of the loci in population and conservation genomic studies.

### Electronic supplementary material

Below is the link to the electronic supplementary material.

Supplementary Material 1: Data S1

Supplementary Material 2: Data S2

Supplementary Material 3: Table S1

Supplementary Material 4: Table S2

Supplementary Material 5: Table S3

Supplementary Material 6: Table S4

Supplementary Material 7: Table S5

Supplementary Material 8: Table S6

Supplementary Material 9: Table S7

Supplementary Material 10: Table S8

Supplementary Material 11: Table S9

Supplementary Material 12: Table S10

Supplementary Material 13: Table S11

Supplementary Material 14: Table S12

Supplementary Material 15: Table S13

Supplementary Material 16: Table S14

Supplementary Material 17: Table S15

Supplementary Material 18: Table S16

Supplementary Material 19: Table S17

Supplementary Material 20: Table S18

Supplementary Material 21: Table S19

Supplementary Material 22: Table S20

Supplementary Material 23: Table S21

Supplementary Material 24: Table S22

Supplementary Material 25: Figure S1

Supplementary Material 26: Figure S2

Supplementary Material 27: Figure S3

Supplementary Material 28: Figure S4

## Data Availability

The datasets supporting the conclusions of this article, including genome assemblies’ accession numbers and all values needed to replicate the study, are included within the article (Data S1, Data S2).
