# Correction: Genome composition and GC content influence loci distribution in reduced representation genomic studies

**DOI:** 10.1186/s12864-024-10385-0

**Published:** 2024-05-10

**Authors:** Carles Galià-Camps, Cinta Pegueroles, Xavier Turon, Carlos Carreras, Marta Pascual

**Affiliations:** 1 Departament de Genètica, Microbiologia I EstadísticaUniversitat de Barcelona, Avinguda Diagonal 643, Barcelona, 08028 Spain; 2 Institut de Recerca de La Biodiversitat (IRBio), Universitat de Barcelona (UB), Barcelona, Spain; 3 Department of Marine Ecology, Centre d’Estudis Avançats de Blanes (CEAB-CSIC), Accés Cala Sant Francesc 14, Blanes, 17300 Spain

**Correction** **:** **BMC Genomics 25, 410 (2024)**

**https://doi.org/10.1186/s12864-024-10312-3**

Following publication of the original article [1], we have been notified that figures 4 and 5 have been swapped. The image shown in Fig. 4 actually belongs to Fig. 5 and vice versa. The original figure captions are correct.

The original article has been corrected.
