# Supplementary material for: Genome composition and GC content influence loci distribution in reduced representation genomic studies
Source: BMC Genomics. 2024 Apr 25;25:410. doi: 10.1186/s12864-024-10312-3 (PMC11046876; doi:10.1186/s12864-024-10312-3)
Supplement: Supplementary file 3 — Supplementary Material 3: Table S1 [file 12864_2024_10312_MOESM3_ESM.pdf]

Table S1: Logarithmic regression equations and their coefficients of determination (R^2) of the percentage of each genomic category (y) with genome size (x). The three taxonomic supergroups include only the species with annotated genomes. Significant p-values are in bold.

Supergroup	Genomic Category	Regression equation	R^2	p-value
**Plants**	**Intergenic**	y=0.806+0.150log(x)	0.87	**<0.001**
	**Intronic**	y=0.095-0.048log(x)	0.69	**0.003**
	**Exonic**	y=0.099-0.103log(x)	0.75	**0.001**
**Protostomes**	**Intergenic**	y=0.495+0.029log(x)	0.03	0.612
	**Intronic**	y=0.469+0.061log(x)	0.17	0.178
	**Exonic**	y=0.035-0.090log(x)	0.68	**0.001**
**Deuterostomes**	**Intergenic**	y=0.481+0.099log(x)	0.49	**<0.001**
	**Intronic**	y=0.439-0.038log(x)	0.13	0.097
	**Exonic**	y=0.080-0.061log(x)	0.80	**<0.001**
