# Supplementary material for: Genome composition and GC content influence loci distribution in reduced representation genomic studies
Source: BMC Genomics. 2024 Apr 25;25:410. doi: 10.1186/s12864-024-10312-3 (PMC11046876; doi:10.1186/s12864-024-10312-3)
Supplement: Supplementary file 4 — Supplementary Material 4: Table S2 [file 12864_2024_10312_MOESM4_ESM.pdf]

Table S2: General Linear Mixed-Effects Models (GLMM) of the percentage of each genomic category. Fixed factors are genomic category (intergenic, intronic, exonic), supergroup (plants, protostomes and deuterostomes) and genome size, using only the species with annotated genomes. Species is considered a random factor. For each factor, we provide their degrees of freedom (DF), chi-square (χ^2) and p-value, and coefficient of determination of the full model and their fixed factors (R^2). Significant p-values are in bold.

Factor	DF	χ2	p-value	R^2 model	R^2 fixed
Intercept	1	56.02	**<0.001**	0.93	0.93
Genomic Category	2	95.38	**<0.001**		
Supergroup	2	2.50	0.286		
Genome Size	1	46.94	**<0.001**		
Genomic Category*Supergroup	4	25.28	**<0.001**		
Genomic Category*Genome Size	2	123.67	**<0.001**		
Supergroup*Genome Size	2	2.46	0.293		
Genomic Category*Supergroup*Genome Size	4	30.57	**<0.001**		
