# Supplementary material for: Genome composition and GC content influence loci distribution in reduced representation genomic studies
Source: BMC Genomics. 2024 Apr 25;25:410. doi: 10.1186/s12864-024-10312-3 (PMC11046876; doi:10.1186/s12864-024-10312-3)
Supplement: Supplementary file 5 — Supplementary Material 5: Table S3 [file 12864_2024_10312_MOESM5_ESM.pdf]

Table S3: Tukey's post-hoc pairwise contrasts for the interaction Genomic Category*Supergroup. The column contrast indicates the factor categories being compared by the post-hoc test and the columns before contrast indicate which factors are being tested (*) or fixed. For each comparison we provide its t-ratio and p-value. Significant p-values are in bold.

Genomic Category	Supergroup	Contrast	t-ratio	p-value
Exonic	*	Plants - Protostomes	0.96	0.999
Exonic	*	Plants - Deuterostomes	-0.02	1.000
Exonic	*	Protostomes - Deuterostomes	-1.04	0.998
Intergenic	*	Plants - Protostomes	6.75	**<0.001**
Intergenic	*	Plants - Deuterostomes	12.36	**<0.001**
Intergenic	*	Protostomes - Deuterostomes	-0.14	1.000
Intronic	*	Plants - Protostomes	-8.20	**<0.001**
Intronic	*	Plants - Deuterostomes	-13.36	**<0.001**
Intronic	*	Protostomes - Deuterostomes	1.11	0.996
*	Plants	Exonic - Intergenic	-24.70	**<0.001**
*	Plants	Exonic - Intronic	-1.06	0.998
*	Plants	Intergenic - Intronic	23.64	**<0.001**
*	Protostomes	Exonic - Intergenic	-8.03	**<0.001**
*	Protostomes	Exonic - Intronic	-7.81	**<0.001**
*	Protostomes	Intergenic - Intronic	0.21	1.000
*	Deuterostomes	Exonic - Intergenic	-23.16	**<0.001**
*	Deuterostomes	Exonic - Intronic	-19.57	**<0.001**
*	Deuterostomes	Intergenic - Intronic	3.59	**0.011**
