# Supplementary material for: Genome composition and GC content influence loci distribution in reduced representation genomic studies
Source: BMC Genomics. 2024 Apr 25;25:410. doi: 10.1186/s12864-024-10312-3 (PMC11046876; doi:10.1186/s12864-024-10312-3)
Supplement: Supplementary file 6 — Supplementary Material 6: Table S4 [file 12864_2024_10312_MOESM6_ESM.pdf]

Table S4: Linear regressions on the number of total and unique loci (y) with genome size (x) considering three models: the 80 genomes altogether (Total model), split by supergroup (plants, protostomes and deuterostomes) and using only the groups with more than six species analyzed (plants, arthropods, fishes, amphibians, mammals and birds). Note that plants, in both the supergroup and group models, include information for the same species but are repeated to facilitate cross comparison in the two models (supergroup and group). For each enzyme, we provide the regression equation, R^2 and p-value. Significant p-values are in bold.

Model	Dataset	Enzyme	TOTAL LOCI			UNIQUE LOCI		
			Regression equation	R^2	p-value	Regression equation	R^2	p-value
Total	80 Genomes	AlfI	y=39516.4+88857.2x	0.64	**<0.001**	y=38512.3+78742.3x	0.56	**<0.001**
		CspCI	y=13363.2+45990.9x	0.82	**<0.001**	y=12914.2+40901.9x	0.73	**<0.001**
		BaeI	y=8141.9+23321.8x	0.84	**<0.001**	y=8646.2+19230.6x	0.76	**<0.001**
Supergroup	Plants	AlfI	y=14100.4+46636.8x	0.84	**<0.001**	y=14474.8+35294.9x	0.89	**<0.001**
		CspCI	y=17441.3+30791.9x	0.95	**<0.001**	y=15360.2+24556.6x	0.87	**<0.001**
		BaeI	y=1351.5+24113.0x	0.95	**<0.001**	y=6601.6+13544.5x	0.74	**<0.001**
	Protostomes	AlfI	y=11517.6+62585.7x	0.64	**<0.001**	y=14691.2+41316.6x	0.50	**0.001**
		CspCI	y=4030.0+35755.0x	0.77	**<0.001**	y=5870.8+23690.5x	0.68	**<0.001**
		BaeI	y=2776.3+34910.2x	0.69	**<0.001**	y=4440.0+23671.1x	0.64	**<0.001**
	Deuterostomes	AlfI	y=91001.7+80646.0x	0.65	**<0.001**	y=89993.0+70892.1x	0.55	**<0.001**
		CspCI	y=25412.8+44657.3x	0.80	**<0.001**	y=26415.4+39487.1x	0.70	**<0.001**
		BaeI	y=11710.2+22039.7x	0.79	**<0.001**	y=11385.8+19545.9x	0.75	**<0.001**
Group	Plants	AlfI	y=14100.4+46636.8x	0.84	**<0.001**	y=14474.8+35294.9x	0.89	**<0.001**
		CspCI	y=17441.3+30791.9x	0.95	**<0.001**	y=15360.2+24556.6x	0.87	**<0.001**
		BaeI	y=1351.5+24113.0x	0.95	**<0.001**	y=6601.6+13544.5x	0.74	**<0.001**
	Arthropods	AlfI	y=11038.6+62495.6x	0.55	**0.010**	y=15815.8+35696.6x	0.35	0.056
		CspCI	y=1000.4+38025.5x	0.84	**<0.001**	y=4011.5+22832.6x	0.75	**0.001**
		BaeI	y=6143.9+21652.7x	0.78	**<0.001**	y=7424.8+12540.4x	0.52	**0.012**
	Fishes	AlfI	y=30979.4+101071.6x	0.83	**<0.001**	y=33929.7+78683.4x	0.65	**<0.001**
		CspCI	y=7737.7+47845.7x	0.79	**<0.001**	y=8690.0+38334.5x	0.58	**0.002**
		BaeI	y=3122.6+26218.0x	0.64	**0.001**	y=4568.8+17930.0x	0.67	**<0.001**
	Amphibians	AlfI	y=113053.3+69735.4x	0.84	**0.013**	y=131130.8+52317.7x	0.73	**0.031**
		CspCI	y=28376.3+36616.9x	0.93	**0.001**	y=38453.5+27438.5x	0.86	**0.007**
		BaeI	y=24232.0+17361.0x	0.94	**0.006**	y=28353.1+12828.4x	0.87	**0.006**
	Mammals	AlfI	y=155748.6+49977.4x	0.20	0.154	y=182134.5+34503.3x	0.13	0.248
		CspCI	y=86161.0+25885.4x	0.24	0.113	y=100447.7+17500.3x	0.16	0.197
		BaeI	y=35476.3+13169.7x	0.21	0.142	y=41153.8+9805.3x	0.15	0.218
	Birds	AlfI	y=-262154.0+460870.9x	0.95	**<0.001**	y=-228179.8+424133.5x	0.85	**<0.001**
		CspCI	y=-3285.3+82365.5x	0.96	**<0.001**	y=6260.4+71250.0x	0.78	**0.001**
		BaeI	y=-60995.7+92940.4x	0.91	**<0.001**	y=-55092.6+86644.1x	0.86	**<0.001**
