# Supplementary material for: Genome composition and GC content influence loci distribution in reduced representation genomic studies
Source: BMC Genomics. 2024 Apr 25;25:410. doi: 10.1186/s12864-024-10312-3 (PMC11046876; doi:10.1186/s12864-024-10312-3)
Supplement: Supplementary file 7 — Supplementary Material 7: Table S5 [file 12864_2024_10312_MOESM7_ESM.pdf]

Table S5: General Linear Mixed-Effects Models of the number of total and unique loci. Three models have been tested including all 80 species combined (Total model), separating species by supergroup (Supergroup model), and separating species by group (Group model). Fixed factors are enzyme (AlfI, CspCI, BaeI), genome size, supergroup (plants, protostomes and deuterostomes) and group (plants, arthropods, fishes, amphibians, mammals and birds). Species are considered a random factor.For each factor we provide the degrees of freedom (DF), chi-square (χ^2) and p-value. For each model we provide the coefficient of determination of the full model and their fixed factors (R^2). Significant p-values are in bold.

			TOTAL LOCI				UNIQUE LOCI			
**Model**	**Factor**	**DF**	**χ2**	**p-value**	**R^2 model**	**R^2 fixed**	**χ2**	**p-value**	**R^2 model**	**R^2 fixed**
Total	Intercept	1	73226.85	**<0.001**	0.93	0.87	54824.25	**<0.001**	0.94	0.84
	Enzyme	2	845.88	**<0.001**			879.81	**<0.001**		
	Genome Size	1	460.58	**<0.001**			350.37	**<0.001**		
	Enzyme*Genome Size	2	8.97	**0.011**			11.87	**0.003**		
Supergroup	Intercept	1	16333.53	**<0.001**	0.97	0.92	12588.44	**<0.001**	0.97	0.90
	Enzyme	2	135.03	**<0.001**			151.35	**<0.001**		
	Supergroup	2	94.41	**<0.001**			100.50	**<0.001**		
	Genome Size	1	78.07	**<0.001**			57.36	**<0.001**		
	Enzyme*Supergroup	4	137.81	**<0.001**			134.13	**<0.001**		
	Enzyme*Genome Size	2	2.27	0.322			0.16	0.922		
	Supergroup*Genome Size	2	0.72	0.697			1.71	0.425		
	Enzyme*Supergroup*Genome Size	4	7.17	0.127			7.28	0.122		
Group	Intercept	1	19875.90	**<0.001**	0.97	0.93	17237.06	**<0.001**	0.97	0.93
	Enzyme	2	154.69	**<0.001**			173.24	**<0.001**		
	Group	5	80.50	**<0.001**			90.91	**<0.001**		
	Genome Size	1	95.00	**<0.001**			78.54	**<0.001**		
	Enzyme*Group	10	98.05	**<0.001**			102.79	**<0.001**		
	Enzyme*Genome Size	2	2.60	0.273			0.19	0.911		
	Group*Genome Size	5	2.91	0.714			4.82	0.438		
	Enzyme*Group*Genome Size	10	4.15	0.940			5.83	0.830		
