# Supplementary material for: Genome composition and GC content influence loci distribution in reduced representation genomic studies
Source: BMC Genomics. 2024 Apr 25;25:410. doi: 10.1186/s12864-024-10312-3 (PMC11046876; doi:10.1186/s12864-024-10312-3)
Supplement: Supplementary file 8 — Supplementary Material 8: Table S6 [file 12864_2024_10312_MOESM8_ESM.pdf]

Table S6: Tukey's post-hoc pairwise contrasts for the interaction Enzyme*Supergroup for total and unique loci. The column contrast indicates the variables being compared with the post-hoc test and the columns before contrast indicate which factors are being tested (*) or fixed. For each comparison we provide its t-ratio and p-value. Significant p-values are in bold.

			TOTAL LOCI		UNIQUE LOCI	
**Supergroup**	**Enzyme**	**Contrast**	**t-ratio**	**p-value**	**t-ratio**	**p-value**
Plants	*	AlfI - CspCI	2.51	0.212	2.52	0.210
Plants	*	AlfI - BaeI	9.73	<0.001	10.59	**<0.001**
Plants	*	CspCI - BaeI	7.22	<0.001	8.08	**<0.001**
Protostomes	*	AlfI - CspCI	4.70	<0.001	4.79	**<0.001**
Protostomes	*	AlfI - BaeI	3.37	0.017	3.47	**0.012**
Protostomes	*	CspCI - BaeI	-1.33	0.975	-1.32	0.976
Deuterostomes	*	AlfI - CspCI	20.41	<0.001	20.35	**<0.001**
Deuterostomes	*	AlfI - BaeI	38.50	<0.001	38.59	**<0.001**
Deuterostomes	*	CspCI - BaeI	18.09	<0.001	18.24	**<0.001**
*	AlfI	Plants - Protostomes	-0.26	1.000	0.23	1.000
*	AlfI	Plants - Deuterostomes	-8.26	<0.001	-8.32	**<0.001**
*	AlfI	Protostomes - Deuterostomes	-5.35	<0.001	-5.96	**<0.001**
*	CspCI	Plants - Protostomes	2.09	0.510	2.41	0.275
*	CspCI	Plants - Deuterostomes	-2.58	0.179	-3.25	**0.027**
*	CspCI	Protostomes - Deuterostomes	-4.15	0.001	-4.97	**<0.001**
*	BaeI	Plants - Protostomes	-2.51	0.212	-2.10	0.499
*	BaeI	Plants - Deuterostomes	-1.69	0.828	-3.03	0.052
*	BaeI	Protostomes - Deuterostomes	1.72	0.812	0.33	1.000
