# Supplementary material for: Genome composition and GC content influence loci distribution in reduced representation genomic studies
Source: BMC Genomics. 2024 Apr 25;25:410. doi: 10.1186/s12864-024-10312-3 (PMC11046876; doi:10.1186/s12864-024-10312-3)
Supplement: Supplementary file 9 — Supplementary Material 9: Table S7 [file 12864_2024_10312_MOESM9_ESM.pdf]

Table S7: Tukey's post-hoc pairwise contrasts for the interaction Enzyme*Group for total and unique loci. The column contrast indicates the variables being compared with the post-hoc test and the columns before contrast indicate which factors are being tested (*) or fixed. For each comparison we provide its t-ratio and p-value. Significant p-values are in bold.

			TOTAL LOCI		UNIQUE LOCI	
**Group**	**Enzyme**	**Contrast**	**t-ratio**	**p-value**	**t-ratio**	**p-value**
Plants	*	AlfI - CspCI	2.66	0.437	2.66	0.435
Plants	*	AlfI - BaeI	10.21	<0.001	11.14	**<0.001**
Plants	*	CspCI - BaeI	7.55	<0.001	8.49	**<0.001**
Arthropods	*	AlfI - CspCI	2.75	0.355	2.79	0.323
Arthropods	*	AlfI - BaeI	1.74	0.996	1.62	0.999
Arthropods	*	CspCI - BaeI	-1.01	1.000	-1.17	1.000
Fishes	*	AlfI - CspCI	10.09	<0.001	10.25	**<0.001**
Fishes	*	AlfI - BaeI	17.66	<0.001	18.67	**<0.001**
Fishes	*	CspCI - BaeI	7.57	<0.001	8.42	**<0.001**
Amphibians	*	AlfI - CspCI	5.26	<0.001	5.30	**<0.001**
Amphibians	*	AlfI - BaeI	8.09	<0.001	8.14	**<0.001**
Amphibians	*	CspCI - BaeI	2.83	0.296	2.84	0.290
Mammals	*	AlfI - CspCI	2.38	0.704	2.37	0.713
Mammals	*	AlfI - BaeI	6.04	<0.001	6.00	**<0.001**
Mammals	*	CspCI - BaeI	3.66	0.025	3.63	**0.027**
Birds	*	AlfI - CspCI	3.14	0.129	3.13	0.133
Birds	*	AlfI - BaeI	3.99	0.008	3.96	**0.009**
Birds	*	CspCI - BaeI	0.85	1.000	0.83	1.000
*	AlfI	Plants - Arthropods	1.29	1.000	1.81	0.992
*	AlfI	Plants - Fishes	-5.82	<0.001	-5.52	**<0.001**
*	AlfI	Plants - Amphibians	-4.36	0.002	-4.81	**<0.001**
*	AlfI	Plants - Mammals	-3.10	0.147	-3.71	**0.022**
*	AlfI	Plants - Birds	-3.61	0.030	-3.71	**0.021**
*	AlfI	Arthropods - Fishes	-4.17	0.004	-4.54	**0.001**
*	AlfI	Arthropods - Amphibians	-4.10	0.005	-4.84	**<0.001**
*	AlfI	Arthropods - Mammals	-3.35	0.070	-4.20	**0.004**
*	AlfI	Arthropods - Birds	-3.90	0.011	-4.25	**0.003**
*	AlfI	Fishes - Amphibians	-0.94	1.000	-1.56	1.000
*	AlfI	Fishes - Mammals	-0.43	1.000	-1.18	1.000
*	AlfI	Fishes - Birds	-2.00	0.955	-2.19	0.864
*	AlfI	Amphibians - Mammals	0.25	1.000	0.03	1.000
*	AlfI	Amphibians - Birds	-1.46	1.000	-1.36	1.000
*	AlfI	Mammals - Birds	-1.53	1.000	-1.30	1.000
*	CspCI	Plants - Arthropods	2.85	0.286	3.33	0.075
*	CspCI	Plants - Fishes	-0.84	1.000	-0.68	1.000
*	CspCI	Plants - Amphibians	-0.90	1.000	-1.48	1.000
*	CspCI	Plants - Mammals	-1.79	0.993	-2.47	0.620
*	CspCI	Plants - Birds	-1.21	1.000	-1.43	1.000

*	CspCI	Arthropods - Fishes	-3.26	0.091	-3.66	**0.026**
*	CspCI	Arthropods - Amphibians	-2.98	0.205	-3.78	**0.017**
*	CspCI	Arthropods - Mammals	-3.45	0.051	-4.32	**0.002**
*	CspCI	Arthropods - Birds	-2.52	0.573	-2.96	0.217
*	CspCI	Fishes - Amphibians	-0.40	1.000	-1.08	1.000
*	CspCI	Fishes - Mammals	-1.40	1.000	-2.16	0.883
*	CspCI	Fishes - Birds	-0.97	1.000	-1.24	1.000
*	CspCI	Amphibians - Mammals	-0.92	1.000	-1.12	1.000
*	CspCI	Amphibians - Birds	-0.73	1.000	-0.69	1.000
*	CspCI	Mammals - Birds	-0.11	1.000	0.05	1.000
*	BaeI	Plants - Arthropods	-0.51	1.000	-0.30	1.000
*	BaeI	Plants - Fishes	-0.79	1.000	-0.68	1.000
*	BaeI	Plants - Amphibians	-1.42	1.000	-2.32	0.765
*	BaeI	Plants - Mammals	-0.83	1.000	-1.84	0.989
*	BaeI	Plants - Birds	-1.80	0.992	-2.18	0.867
*	BaeI	Arthropods - Fishes	0.13	1.000	-0.03	1.000
*	BaeI	Arthropods - Amphibians	-0.56	1.000	-1.36	1.000
*	BaeI	Arthropods - Mammals	-0.27	1.000	-1.22	1.000
*	BaeI	Arthropods - Birds	-1.36	1.000	-1.81	0.992
*	BaeI	Fishes - Amphibians	-0.96	1.000	-1.92	0.977
*	BaeI	Fishes - Mammals	-0.47	1.000	-1.53	1.000
*	BaeI	Fishes - Birds	-1.58	1.000	-2.00	0.957
*	BaeI	Amphibians - Mammals	0.24	1.000	-0.03	1.000
*	BaeI	Amphibians - Birds	-1.06	1.000	-1.02	1.000
*	BaeI	Mammals - Birds	-1.15	1.000	-0.94	1.000
