# Supplementary material for: Genome composition and GC content influence loci distribution in reduced representation genomic studies
Source: BMC Genomics. 2024 Apr 25;25:410. doi: 10.1186/s12864-024-10312-3 (PMC11046876; doi:10.1186/s12864-024-10312-3)
Supplement: Supplementary file 10 — Supplementary Material 10: Table S8 [file 12864_2024_10312_MOESM10_ESM.pdf]

Table S8: General Linear Mixed-Effects Models (GLMM) of the percentage of unique loci (in relation to the total number of loci) for the 80 genomes. Three models have been tested including all species combined (Total model), separating species by supergroup (Supergroup model), and separating species by group (Group model). Fixed factors are: enzyme (AlfI, CspCI, BaeI), genome size, supergroup (plants, protostomes and deuterostomes) and group (plants, arthropods, fishes, amphibians, mammals and birds). Species are considered a random factor. For each factor we provide the degrees of freedom (DF), chi-square (χ^2) and p-value. For each model we provide the coefficient of determination of the full model and their fixed factors (R^2). Significant p-values are in bold.

**Model**	**Factor**	**DF**	**χ2**	**p-value**	**R^2 model**	**R^2 fixed**
Total	Enzyme	2	0.98	0.614	0.85	0.00
	Genome Size	1	0.24	0.623		
	Enzyme*Genome Size	2	1.45	0.485		
Supergroup	Intercept	1	937.30	**<0.001**	0.91	0.23
	Enzyme	2	1.17	0.558		
	Genome Size	1	0.88	0.349		
	Supergroup	2	26.22	**<0.001**		
	Enzyme*Genome Size	2	6.86	**0.032**		
	Enzyme*Supergroup	4	1.03	0.906		
	Genome Size*Supergroup	2	3.90	0.142		
	Enzyme*Genome Size*Supergroup	4	5.23	0.265		
Group	Intercept	1	1413.66	**<0.001**	0.94	0.49
	Enzyme	2	1.65	0.438		
	Genome Size	1	1.32	0.250		
	Group	5	28.66	**<0.001**		
	Enzyme*Genome Size	2	9.71	**0.008**		
	Enzyme*Group	10	7.20	0.706		
	Genome Size*Group	5	5.33	0.377		
	Enzyme*Genome Size*Group	10	5.93	0.821		
