# Supplementary material for: Genome composition and GC content influence loci distribution in reduced representation genomic studies
Source: BMC Genomics. 2024 Apr 25;25:410. doi: 10.1186/s12864-024-10312-3 (PMC11046876; doi:10.1186/s12864-024-10312-3)
Supplement: Supplementary file 11 — Supplementary Material 11: Table S9 [file 12864_2024_10312_MOESM11_ESM.pdf]

Table S9: Tukey's post-hoc pairwise contrasts between supergroups (plants, protostomes and deuterostomes) on the percentage of unique loci. For each comparison, we provide its t-ratio and p-value. Significant p-values are in bold.

Contrast	t-ratio	p-value
Plants - Protostomes	1.22	0.539
Plants - Deuterostomes	-4.08	**<0.001**
Protostomes - Deuterostomes	-4.18	**<0.001**
