# Supplementary material for: Genome composition and GC content influence loci distribution in reduced representation genomic studies
Source: BMC Genomics. 2024 Apr 25;25:410. doi: 10.1186/s12864-024-10312-3 (PMC11046876; doi:10.1186/s12864-024-10312-3)
Supplement: Supplementary file 12 — Supplementary Material 12: Table S10 [file 12864_2024_10312_MOESM12_ESM.pdf]

Table S10: Tukey's post-hoc pairwise comparisons between groups (plants, arthropods, fishes, amphibians, mammals and birds) on the percentage of unique loci. For each comparison, we provide its t-ratio and p-value. Significant p-values are in bold.

Contrast	t-ratio	p-value
Plants - Arthropods	1.47	0.909
Plants - Fishes	-0.41	1.000
Plants - Amphibians	-2.57	0.179
Plants - Mammals	-3.76	**0.006**
Plants - Birds	-1.28	0.968
Arthropods - Fishes	-1.67	0.796
Arthropods - Amphibians	-3.00	0.060
Arthropods - Mammals	-3.99	**0.003**
Arthropods - Birds	-1.89	0.627
Fishes - Amphibians	-2.32	0.306
Fishes - Mammals	-3.57	**0.011**
Fishes - Birds	-1.17	0.986
Amphibians - Mammals	-1.49	0.898
Amphibians - Birds	-0.07	1.000
Mammals - Birds	0.87	0.999
