# Supplementary material for: Genome composition and GC content influence loci distribution in reduced representation genomic studies
Source: BMC Genomics. 2024 Apr 25;25:410. doi: 10.1186/s12864-024-10312-3 (PMC11046876; doi:10.1186/s12864-024-10312-3)
Supplement: Supplementary file 13 — Supplementary Material 13: Table S11 [file 12864_2024_10312_MOESM13_ESM.pdf]

Table S11: Linear regressions between the percentage of unique loci in a genomic category (y) and the percentage of the same genomic category in the genome (x). The regressions are carried out only considering the annotated genomes for each supergroup (plants, protostomes, deuterostomes), genomic category (intergenic, intronic, exonic), and enzyme (AlfI, CspCI, BaeI) independently. For each combination, we provide the regression equation, the coefficient of determination of the model (R^2) and p-values. Significant p-values are given in bold.

Supergroup	Genomic Category	Enzyme	Regression equation	R^2	p-value
**Plants**	**Intergenic**	**AlfI**	y=-0.164+1.186x	0.94	**<0.001**
		**CspCI**	y=-0.293+1.165x	0.97	**<0.001**
		**BaeI**	y=-0.273+1.146x	0.94	**<0.001**
	**Intronic**	**AlfI**	y=0.001+0.735x	0.45	**0.033**
		**CspCI**	y=-0.009+0.996x	0.53	**0.017**
		**BaeI**	y=-0.020+0.955x	0.67	**0.004**
	**Exonic**	**AlfI**	y=0.139+1.465x	0.92	**<0.001**
		**CspCI**	y=0.098+1.482x	0.95	**<0.001**
		**BaeI**	y=0.089+1.384x	0.94	**<0.001**
**Protostomes**	**Intergenic**	**AlfI**	y=-0.104+1.029x	0.98	**<0.001**
		**CspCI**	y=-0.108+1.032x	0.95	**<0.001**
		**BaeI**	y=-0.092+1.013x	0.94	**<0.001**
	**Intronic**	**AlfI**	y=0.088+0.849x	0.84	**<0.001**
		**CspCI**	y=0.112+0.827x	0.75	**<0.001**
		**BaeI**	y=0.072+0.897x	0.88	**<0.001**
	**Exonic**	**AlfI**	y=0.064+1.433x	0.87	**<0.001**
		**CspCI**	y=0.065+1.592x	0.80	**<0.001**
		**BaeI**	y=0.045+1.558x	0.90	**<0.001**
**Deuterostomes**	**Intergenic**	**AlfI**	y=-0.064+0.940x	0.82	**<0.001**
		**CspCI**	y=-0.079+0.987x	0.84	**<0.001**
		**BaeI**	y=-0.082+0.971x	0.84	**<0.001**
	**Intronic**	**AlfI**	y=0.089+0.722x	0.58	**<0.001**
		**CspCI**	y=0.139+0.634x	0.49	**<0.001**
		**BaeI**	y=0.102+0.699x	0.56	**<0.001**
	**Exonic**	**AlfI**	y=0.014+1.724x	0.93	**<0.001**
		**CspCI**	y=0.002+2.055x	0.95	**<0.001**
		**BaeI**	y=0.014+1.848x	0.92	**<0.001**
