# Supplementary material for: Genome composition and GC content influence loci distribution in reduced representation genomic studies
Source: BMC Genomics. 2024 Apr 25;25:410. doi: 10.1186/s12864-024-10312-3 (PMC11046876; doi:10.1186/s12864-024-10312-3)
Supplement: Supplementary file 14 — Supplementary Material 14: Table S12 [file 12864_2024_10312_MOESM14_ESM.pdf]

Table S12: General Linear Mixed-Effects Models of the ratio between the percentage of unique loci in a genomic category and the percentage of genome in the same genomic category. Factors considered for evaluation in the GLMM are enzyme (AlfI, CspCI, BaeI), supergroup (plants, protostomes, deuterostomes), and genomic category (intergenic, intronic, exonic) and their pairwise interactions. For each factor we provide the degrees of freedom (DF), chi-square (χ^2) and p-value. We provide the coefficient of determination (R^2) of the full model and only considering the fixed factors. Significant p-values are in bold.

Factor	DF	χ2	p-value	R^2 model	R^2 fixed
Intercept	1	1295.55	**<0.001**	0.79	0.77
Enzyme	2	18.65	**<0.001**		
Supergroup	2	84.76	**<0.001**		
Genomic Category	2	363.66	**<0.001**		
Enzyme*Supergroup	4	15.67	**0.003**		
Enzyme*Genomic Category	4	18.32	**0.001**		
Supergroup*Genomic Category	4	94.62	**<0.001**		
Enzyme*Supergroup*Genomic Category	8	14.65	0.066		
