# Supplementary material for: Genome composition and GC content influence loci distribution in reduced representation genomic studies
Source: BMC Genomics. 2024 Apr 25;25:410. doi: 10.1186/s12864-024-10312-3 (PMC11046876; doi:10.1186/s12864-024-10312-3)
Supplement: Supplementary file 15 — Supplementary Material 15: Table S13 [file 12864_2024_10312_MOESM15_ESM.pdf]

Table S13: Tukey's post-hoc pairwise contrasts for the interactions Supergroup*Enzyme, Genomic Category*Enzyme and Genomic Category*Supergroup for the ratio between the percentage of loci in a genomic category and the percentage of the same genomic category in the genome. The column contrast indicates the variables being compared with the post-hoc test and the columns before contrast indicate which factors are being tested (*) or fixed. For each comparison we provide its t-ratio and p-value. Significant p-values are in bold.

Interaction	Supergroup	Enzyme	Contrast	t-ratio	p-value
Supergroup * Enzyme	Plants	*	AlfI - CspCI	0.59	1.000
	Plants	*	AlfI - BaeI	1.66	0.843
	Plants	*	CspCI - BaeI	1.08	0.998
	Protostomes	*	AlfI - CspCI	-0.79	1.000
	Protostomes	*	AlfI - BaeI	0.09	1.000
	Protostomes	*	CspCI - BaeI	0.88	1.000
	Deuterostomes	*	AlfI - CspCI	-0.30	1.000
	Deuterostomes	*	AlfI - BaeI	-0.04	1.000
	Deuterostomes	*	CspCI - BaeI	0.26	1.000
	*	AlfI	Plants - Protostomes	1.74	0.790
	*	AlfI	Plants - Deuterostomes	2.77	0.107
	*	AlfI	Protostomes - Deuterostomes	0.86	1.000
	*	CspCI	Plants - Protostomes	0.50	1.000
	*	CspCI	Plants - Deuterostomes	1.93	0.641
	*	CspCI	Protostomes - Deuterostomes	1.45	0.944
	*	BaeI	Plants - Protostomes	0.24	1.000
	*	BaeI	Plants - Deuterostomes	0.97	0.999
	*	BaeI	Protostomes - Deuterostomes	0.74	1.000
	**Genomic Category**	**Enzyme**	**Contrast**	**t-ratio**	**p-value**
Genomic Category * Enzyme	Exonic	*	AlfI - CspCI	1.51	0.922
	Exonic	*	AlfI - BaeI	2.91	0.068
	Exonic	*	CspCI - BaeI	1.40	0.960
	Intergenic	*	AlfI - CspCI	-0.62	1.000
	Intergenic	*	AlfI - BaeI	-0.66	1.000
	Intergenic	*	CspCI - BaeI	-0.04	1.000
	Intronic	*	AlfI - CspCI	-1.28	0.983
	Intronic	*	AlfI - BaeI	-0.28	1.000
	Intronic	*	CspCI - BaeI	0.99	0.999
	*	AlfI	Exonic - Intergenic	21.52	**<0.001**
	*	AlfI	Exonic - Intronic	18.60	**<0.001**
	*	AlfI	Intergenic - Intronic	-2.92	0.066
	*	CspCI	Exonic - Intergenic	19.39	**<0.001**
	*	CspCI	Exonic - Intronic	15.81	**<0.001**
	*	CspCI	Intergenic - Intronic	-3.57	**0.007**
	*	BaeI	Exonic - Intergenic	17.95	**<0.001**
	*	BaeI	Exonic - Intronic	15.41	**<0.001**
	*	BaeI	Intergenic - Intronic	-2.54	0.189

	Genomic Category	Supergroup	Contrast	t-ratio	p-value
Genomic Category * Supergroup	Exonic	*	Plants - Protostomes	8.26	**<0.001**
	Exonic	*	Plants - Deuterostomes	9.77	**<0.001**
	Exonic	*	Protostomes - Deuterostomes	0.52	1.000
	Intergenic	*	Plants - Protostomes	-1.66	0.846
	Intergenic	*	Plants - Deuterostomes	-2.12	0.474
	Intergenic	*	Protostomes - Deuterostomes	-0.28	1.000
	Intronic	*	Plants - Protostomes	-4.12	**0.001**
	Intronic	*	Plants - Deuterostomes	-1.97	0.604
	Intronic	*	Protostomes - Deuterostomes	2.82	0.093
	*	Plants	Exonic - Intergenic	24.34	**<0.001**
	*	Plants	Exonic - Intronic	22.52	**<0.001**
	*	Plants	Intergenic - Intronic	-1.81	0.733
	*	Protostomes	Exonic - Intergenic	15.21	**<0.001**
	*	Protostomes	Exonic - Intronic	10.38	**<0.001**
	*	Protostomes	Intergenic - Intronic	-4.82	**<0.001**
	*	Deuterostomes	Exonic - Intergenic	19.55	**<0.001**
	*	Deuterostomes	Exonic - Intronic	17.07	**<0.001**
	*	Deuterostomes	Intergenic - Intronic	-2.48	0.220
