# Supplementary material for: Genome composition and GC content influence loci distribution in reduced representation genomic studies
Source: BMC Genomics. 2024 Apr 25;25:410. doi: 10.1186/s12864-024-10312-3 (PMC11046876; doi:10.1186/s12864-024-10312-3)
Supplement: Supplementary file 16 — Supplementary Material 16: Table S14 [file 12864_2024_10312_MOESM16_ESM.pdf]

Table S14: General Linear Mixed-Effects Models of the percentage of selected unique loci after secondary reduction including the 80 genomes**.** Three models have been tested including all species combined (Total model), separating species by supergroup (Supergroup model), and separating species by group (Group model). Fixed factors are enzyme (AlfI, CspCI, BaeI), selection (S, W), genome size, supergroup (plants, protostomes, deuterostomes), and group (plants, arthropods, fishes, amphibians, mammals and birds). Species are considered a random factor. For each factor we provide the degrees of freedom (DF), chi-square (χ^2) and p-value. For each model we provide the coefficient of determination of the full model and their fixed factors (R^2). Significant p-values are in bold.

Model	Factor	DF	χ2	p-value	R^2 model	R^2 fixed
Total	Intercept	1	18650.34	**<0.001**	0.87	0.87
	Enzyme	2	22.41	**<0.001**		
	Selection	1	829.63	**<0.001**		
	Genome Size	1	4.60	**0.032**		
	Enzyme*Selection	2	38.72	**<0.001**		
	Enzyme*Genome Size	2	5.03	0.081		
	Selection*Genome Size	1	7.04	**0.008**		
	Enzyme*Selection*Genome Size	2	8.91	**0.012**		
Supergroup	Intercept	1	3428.80	**<0.001**	0.90	0.90
	Enzyme	2	5.83	0.054		
	Selection	1	253.03	**<0.001**		
	Supergroup	2	23.79	**<0.001**		
	Genome Size	1	2.96	0.086		
	Enzyme*Selection	2	9.60	**0.008**		
	Enzyme*Supergroup	4	2.71	0.608		
	Selection*Supergroup	2	50.01	**<0.001**		
	Enzyme*Genome Size	2	1.22	0.544		
	Selection*Genome Size	1	5.52	**0.019**		
	Supergroup*Genome Size	2	17.74	**<0.001**		
	Enzyme*Selection*Supergroup	4	4.71	0.318		
	Enzyme*Selection*Genome Size	2	2.41	0.300		
	Enzyme*Supergroup*Genome Size	4	0.56	0.967		
	Selection*Supergroup*Genome Size	2	36.78	**<0.001**		
	Enzyme*Selection*Supergroup*Genome Size	4	1.10	0.894		
Group	Intercept	1	5059.08	**<0.001**	0.92	0.92
	Enzyme	2	8.61	**0.014**		
	Selection	1	373.33	**<0.001**		
	Group	5	8.59	0.127		
	Genome Size	1	4.36	**0.037**		
	Enzyme*Selection	2	14.16	**0.001**		
	Enzyme*Group	10	4.38	0.929		
	Selection*Group	5	18.71	**0.002**		
	Enzyme*Genome Size	2	1.80	0.407		

	Selection*Genome Size	1	8.15	**0.004**		
	Group*Genome Size	5	19.54	**0.002**		
	Enzyme*Selection*Group	10	8.21	0.609		
	Enzyme*Selection*Genome Size	2	3.55	0.169		
	Enzyme*Group*Genome Size	10	0.82	1.000		
	Selection*Group*Genome Size	5	40.14	**<0.001**		
	Enzyme*Selection*Group*Genome Size	10	1.72	0.998		
