# Supplementary material for: Genome composition and GC content influence loci distribution in reduced representation genomic studies
Source: BMC Genomics. 2024 Apr 25;25:410. doi: 10.1186/s12864-024-10312-3 (PMC11046876; doi:10.1186/s12864-024-10312-3)
Supplement: Supplementary file 17 — Supplementary Material 17: Table S15 [file 12864_2024_10312_MOESM17_ESM.pdf]

Table S15: Tukey's post-hoc pairwise contrasts for the interaction Selection*Enzyme for the percentage of selected unique loci after secondary reduction on the Total model. The column contrast indicates the variables being compared with the post-hoc test and the columns before contrast indicate which factors are being tested (*) or fixed. For each comparison we provide its t-ratio and p-value. Significant p-values are in bold.

Selection	Enzyme	Contrast	t-ratio	p-value
S	*	AlfI - CspCI	1.57	0.679
S	*	AlfI - BaeI	5.00	**<0.001**
S	*	CspCI - BaeI	3.43	**0.006**
W	*	AlfI - CspCI	-1.89	0.427
W	*	AlfI - BaeI	-4.45	**<0.001**
W	*	CspCI - BaeI	-2.56	0.094
*	AlfI	S - W	-25.97	**<0.001**
*	CspCI	S - W	-29.42	**<0.001**
*	BaeI	S - W	-35.41	**<0.001**
