# Supplementary material for: Genome composition and GC content influence loci distribution in reduced representation genomic studies
Source: BMC Genomics. 2024 Apr 25;25:410. doi: 10.1186/s12864-024-10312-3 (PMC11046876; doi:10.1186/s12864-024-10312-3)
Supplement: Supplementary file 18 — Supplementary Material 18: Table S16 [file 12864_2024_10312_MOESM18_ESM.pdf]

Table S16: Tukey's post-hoc pairwise contrasts for the interactions Selection*Enzyme and Selection*Supergroup for the percentage of selected unique loci after secondary reduction on the Supergroup model. The column contrast indicates the variables being compared with the post-hoc test and the columns before contrast indicate which factors are being tested (*) or fixed. For each comparison we provide its t-ratio and p-value. Significant p-values are in bold.

Interaction	Selection	Enzyme	Contrast	t-ratio	p-value
Selection*Enzyme	S	*	AlfI - CspCI	0.34	1.000
	S	*	AlfI - BaeI	2.55	0.096
	S	*	CspCI - BaeI	2.22	0.221
	W	*	AlfI - CspCI	-0.70	0.997
	W	*	AlfI - BaeI	-2.27	0.197
	W	*	CspCI - BaeI	-1.56	0.680
	*	AlfI	S - W	-23.47	**<0.001**
	*	CspCI	S - W	-24.51	**<0.001**
	*	BaeI	S - W	-28.29	**<0.001**
	**Selection**	**Supergroup**	**Contrast**	**t-ratio**	**p-value**
Selection*Supergroup	S	*	Plants - Protostomes	3.90	**0.001**
	S	*	Plants - Deuterostomes	-3.07	**0.021**
	S	*	Protostomes - Deuterostomes	-6.54	**<0.001**
	W	*	Plants - Protostomes	-3.90	**0.001**
	W	*	Plants - Deuterostomes	3.76	**0.002**
	W	*	Protostomes - Deuterostomes	7.01	**<0.001**
	*	Plants	S - W	-26.25	**<0.001**
	*	Protostomes	S - W	-23.51	**<0.001**
	*	Deuterostomes	S - W	-46.13	**<0.001**
