# Supplementary material for: Genome composition and GC content influence loci distribution in reduced representation genomic studies
Source: BMC Genomics. 2024 Apr 25;25:410. doi: 10.1186/s12864-024-10312-3 (PMC11046876; doi:10.1186/s12864-024-10312-3)
Supplement: Supplementary file 19 — Supplementary Material 19: Table S17 [file 12864_2024_10312_MOESM19_ESM.pdf]

Table S17: Tukey's post-hoc pairwise contrasts for the interactions Selection*Enzyme and Selection*Group for the percentage of selected unique loci after secondary reduction on the Group model. The column contrast indicates the variables being compared with the post-hoc test and the columns before contrast indicate which factors are being tested (*) or fixed. For each comparison we provide its t-ratio and p-value. Significant p-values are in bold.

Interaction	Selection	Enzyme	Contrast	t-ratio	p-value
Selection*Enzyme	S	*	AlfI - CspCI	0.99	0.970
	S	*	AlfI - BaeI	2.41	0.142
	S	*	CspCI - BaeI	1.41	0.789
	W	*	AlfI - CspCI	-1.14	0.930
	W	*	AlfI - BaeI	-2.18	0.242
	W	*	CspCI - BaeI	-1.04	0.959
	*	AlfI	S - W	-11.32	**<0.001**
	*	CspCI	S - W	-13.45	**<0.001**
	*	BaeI	S - W	-15.91	**<0.001**
	**Selection**	**Group**	**Contrast**	**t-ratio**	**p-value**
Selection*Group	S	*	Plants - Arthropods	1.80	0.937
	S	*	Plants - Fishes	0.38	1.000
	S	*	Plants - Amphibians	1.14	1.000
	S	*	Plants - Mammals	-3.81	**0.007**
	S	*	Plants - Birds	-2.58	0.323
	S	*	Arthropods - Fishes	-1.61	0.985
	S	*	Arthropods - Amphibians	-0.70	1.000
	S	*	Arthropods - Mammals	-4.26	**0.001**
	S	*	Arthropods - Birds	-3.22	0.053
	S	*	Fishes - Amphibians	0.91	1.000
	S	*	Fishes - Mammals	-3.98	**0.004**
	S	*	Fishes - Birds	-2.68	0.252
	S	*	Amphibians - Mammals	-3.97	**0.004**
	S	*	Amphibians - Birds	-2.91	0.138
	S	*	Mammals - Birds	-0.25	1.000
	W	*	Plants - Arthropods	-1.68	0.973
	W	*	Plants - Fishes	0.32	1.000
	W	*	Plants - Amphibians	-0.88	1.000
	W	*	Plants - Mammals	3.80	**0.007**
	W	*	Plants - Birds	2.60	0.305
	W	*	Arthropods - Fishes	1.83	0.923
	W	*	Arthropods - Amphibians	0.77	1.000
	W	*	Arthropods - Mammals	4.17	**0.002**
	W	*	Arthropods - Birds	3.18	0.060
	W	*	Fishes - Amphibians	-1.07	1.000
	W	*	Fishes - Mammals	3.65	**0.012**
	W	*	Fishes - Birds	2.51	0.372
	W	*	Amphibians - Mammals	3.80	**0.007**

	W	*	Amphibians - Birds	2.82	0.176
	W	*	Mammals - Birds	0.27	1.000
	*	Plants	S - W	-31.28	**<0.001**
	*	Arthropods	S - W	-14.24	**<0.001**
	*	Fishes	S - W	-31.09	**<0.001**
	*	Amphibians	S - W	-15.82	**<0.001**
	*	Mammals	S - W	-4.98	**<0.001**
	*	Birds	S - W	-2.48	0.392
