# Supplementary material for: Genome composition and GC content influence loci distribution in reduced representation genomic studies
Source: BMC Genomics. 2024 Apr 25;25:410. doi: 10.1186/s12864-024-10312-3 (PMC11046876; doi:10.1186/s12864-024-10312-3)
Supplement: Supplementary file 20 — Supplementary Material 20: Table S18 [file 12864_2024_10312_MOESM20_ESM.pdf]

Table S18: Linear regressions on the number of percentage of selected loci by W and S adaptors (y) with GC content (x) considering independently each enzyme (AlfI, CspCI, BaeI) used on each supergroup (plants, protostomes and deuterostomes). For each enzyme, we provide the regression equation, R^2 and p-value. Significant p-values are in bold.

		**W-selection**			**S-selection**		
**Supergroup**	**Enzyme**	**Regression equation**	**R^2**	**p-value**	**Regression equation**	**R^2**	**p-value**
Plants	AlfI	y=0.639-0.008x	0.90	**0.000**	y=-0.1+0.007x	0.93	**0.000**
	CspCI	y=0.545-0.005x	0.73	**0.000**	y=-0.033+0.006x	0.83	**0.000**
	BaeI	y=0.523-0.004x	0.62	**0.001**	y=-0.002+0.004x	0.80	**0.000**
Protostomes	AlfI	y=0.548-0.006x	0.45	**0.003**	y=0.028+0.004x	0.38	**0.008**
	CspCI	y=0.534-0.006x	0.44	**0.004**	y=0.036+0.004x	0.39	**0.008**
	BaeI	y=0.536-0.006x	0.47	**0.002**	y=0.023+0.004x	0.47	**0.003**
Deuterostomes	AlfI	y=0.562-0.006x	0.36	**0.000**	y=-0.018+0.005x	0.33	**0.000**
	CspCI	y=0.526-0.005x	0.45	**0.000**	y=0.029+0.004x	0.41	**0.000**
	BaeI	y=0.522-0.005x	0.46	**0.000**	y=0.012+0.004x	0.32	**0.000**
