# Supplementary material for: Genome composition and GC content influence loci distribution in reduced representation genomic studies
Source: BMC Genomics. 2024 Apr 25;25:410. doi: 10.1186/s12864-024-10312-3 (PMC11046876; doi:10.1186/s12864-024-10312-3)
Supplement: Supplementary file 21 — Supplementary Material 21: Table S19 [file 12864_2024_10312_MOESM21_ESM.pdf]

Table S19: Linear regressions between the percentage of unique loci in a genomic category (y) and the percentage of the same category in the genome (x). Each supergroup (plants, protostomes and deuterostomes), genomic category (intergenic, intronic, exonic), enzyme (AlfI, CspCI, BaeI) and selection (W-selection, S-selection) has been considered independently. For each combination, we provide the regression equation, the coefficient of determination (R^2) and p-values. Significant p-values are given in bold.

Supergroup	Genomic Category	Enzyme	W-selection			S-selection		
			Regression equation	R^2	p-value	Regression equation	R^2	p-value
Plants	Intergenic	AlfI	y=-0.362+1.167x	0.93	**<0.001**	y=-0.407+1.235x	0.95	**<0.001**
		CspCI	y=-0.226+1.091x	0.95	**<0.001**	y=-0.358+1.232x	0.97	**<0.001**
		BaeI	y=-0.222+1.091x	0.92	**<0.001**	y=-0.349+1.226x	0.96	**<0.001**
	Intronic	AlfI	y=0.083+0.701x	0.51	**0.020**	y=0.065+0.520x	0.39	0.056
		CspCI	y=0.061+0.648x	0.58	**0.011**	y=0.055+0.364x	0.33	0.081
		BaeI	y=0.061+0.814x	0.70	**0.003**	y=0.054+0.517x	0.50	**0.023**
	Exonic	AlfI	y=0.133+1.405x	0.92	**<0.001**	y=0.143+1.566x	0.92	**<0.001**
		CspCI	y=0.09+1.351x	0.95	**<0.001**	y=0.114+1.635x	0.93	**<0.001**
		BaeI	y=0.078+1.263x	0.94	**<0.001**	y=0.101+1.580x	0.94	**<0.001**
Protostomes	Intergenic	AlfI	y=-0.114+1.064x	0.97	**<0.001**	y=-0.090+0.987x	0.98	**<0.001**
		CspCI	y=-0.099+1.025x	0.94	**<0.001**	y=-0.109+1.013x	0.95	**<0.001**
		BaeI	y=-0.109+1.087x	0.96	**<0.001**	y=-0.059+0.910x	0.91	**<0.001**
	Intronic	AlfI	y=-0.019+1.01x	0.82	**<0.001**	y=-0.026+0.929x	0.80	**<0.001**
		CspCI	y=-0.008+0.942x	0.78	**<0.001**	y=-0.015+0.833x	0.67	**0.001**
		BaeI	y=-0.012+1x	0.90	**<0.001**	y=-0.039+0.937x	0.80	**<0.001**
	Exonic	AlfI	y=0.037+1.459x	0.85	**<0.001**	y=0.100+1.372x	0.82	**<0.001**
		CspCI	y=0.045+1.552x	0.84	**<0.001**	y=0.108+1.570x	0.74	**<0.001**
		BaeI	y=0.023+1.431x	0.90	**<0.001**	y=0.096+1.515x	0.83	**<0.001**
Deuterostomes	Intergenic	AlfI	y=-0.07+0.967x	0.84	**<0.001**	y=-0.061+0.917x	0.80	**<0.001**
		CspCI	y=-0.088+1.013x	0.86	**<0.001**	y=-0.079+0.972x	0.83	**<0.001**
		BaeI	y=-0.079+0.978x	0.85	**<0.001**	y=-0.095+0.973x	0.81	**<0.001**
	Intronic	AlfI	y=0.115+0.848x	0.65	**<0.001**	y=0.136+0.745x	0.47	**<0.001**
		CspCI	y=0.114+0.81x	0.55	**<0.001**	y=0.127+0.731x	0.41	**0.001**
		BaeI	y=0.115+0.841x	0.62	**<0.001**	y=0.122+0.764x	0.49	**<0.001**
	Exonic	AlfI	y=0.004+1.595x	0.94	**<0.001**	y=0.026+1.911x	0.91	**<0.001**
		CspCI	y=-0.006+1.977x	0.96	**<0.001**	y=0.011+2.249x	0.93	**<0.001**
		BaeI	y=0.003+1.717x	0.94	**<0.001**	y=0.032+1.998x	0.86	**<0.001**
