# Supplementary material for: Genome composition and GC content influence loci distribution in reduced representation genomic studies
Source: BMC Genomics. 2024 Apr 25;25:410. doi: 10.1186/s12864-024-10312-3 (PMC11046876; doi:10.1186/s12864-024-10312-3)
Supplement: Supplementary file 22 — Supplementary Material 22: Table S20 [file 12864_2024_10312_MOESM22_ESM.pdf]

Table S20: General Linear Mixed-Effects Models for the ratio between the percentage of loci in a genomic category after selection and the percentage of the same genomic category in the genome using the annotated genomes**.** Fixed factors are enzyme (AlfI, CspCI, BaeI), selection (S, W), supergroup (plants, protostomes, deuterostomes), and genomic category (intergenic, intronic, exonic). For each factor we provide the degrees of freedom (DF), chi-square (χ^2) and p-value. We provide the coefficients of determination of the full model and of only their fixed factors (R^2). Significant p-values are in bold.

**Factor**	**DF**	**χ2**	**p-value**	**R^2 model**	**R^2 fixed**
Intercept	1	1151.05	**<0.001**	0.86	0.75
Enzyme	2	15.45	**<0.001**		
Selection	1	2.34	0.126		
Supergroup	2	52.81	**<0.001**		
Genomic Category	2	467.27	**<0.001**		
Enzyme*Selection	2	2.23	0.328		
Enzyme*Supergroup	4	18.95	**0.001**		
Enzyme*Genomic Category	4	13.93	**0.008**		
Selection*Supergroup	2	6.41	**0.041**		
Selection*Genomic Category	2	9.70	**0.008**		
Supergroup*Genomic Category	4	74.71	**<0.001**		
Selection*Supergroup*Genomic Category	4	4.70	0.319		
Enzyme*Selection*Supergroup	4	3.24	0.518		
Enzyme*Supergroup*Genomic Category	8	16.06	**0.042**		
Enzyme*Selection*Genomic Category	4	1.96	0.744		
Enzyme*Selection*Supergroup*Genomic Category	8	2.82	0.945		
