# Supplementary material for: Genome composition and GC content influence loci distribution in reduced representation genomic studies
Source: BMC Genomics. 2024 Apr 25;25:410. doi: 10.1186/s12864-024-10312-3 (PMC11046876; doi:10.1186/s12864-024-10312-3)
Supplement: Supplementary file 23 — Supplementary Material 23: Table S21 [file 12864_2024_10312_MOESM23_ESM.pdf]

Table S21: Tukey's post-hoc pairwise contrasts on the 2-way interactions (selection*genomic category, selection*supergroup). The column contrast indicates the variables being compared with the post-hoc test and the first two columns indicate which factors are fixed (the factor being tested is represented with an asterisk). For each comparison we provide its p-value. Significant p-values are in bold.

Interaction	Genomic Category	Selection	Contrast	t-ratio	p-value
Selection*Genomic Category	Exonic	*	S - W	12.27	**<0.001**
	Intergenic	*	S - W	-1.12	0.935
	Intronic	*	S - W	-4.96	**<0.001**
	*	S	Exonic - Intergenic	48.18	**<0.001**
	*	S	Exonic - Intronic	38.80	**<0.001**
	*	S	Intergenic - Intronic	-9.38	**<0.001**
	*	W	Exonic - Intergenic	34.79	**<0.001**
	*	W	Exonic - Intronic	21.57	**<0.001**
	*	W	Intergenic - Intronic	-13.22	**<0.001**
	**Supergroup**	**Selection**	**Contrast**	**t-ratio**	**p-value**
Selection*Supergroup	Plants	*	S - W	-0.28	1.000
	Protostomes	*	S - W	2.41	0.137
	Deuterostomes	*	S - W	5.17	**<0.001**
	*	S	Plants - Protostomes	2.76	0.072
	*	S	Plants - Deuterostomes	2.19	0.267
	*	S	Protostomes - Deuterostomes	-0.97	0.975
	*	W	Plants - Protostomes	3.66	**0.006**
	*	W	Plants - Deuterostomes	3.72	**0.005**
	*	W	Protostomes - Deuterostomes	-0.41	1.000
