# Supplementary material for: Genome composition and GC content influence loci distribution in reduced representation genomic studies
Source: BMC Genomics. 2024 Apr 25;25:410. doi: 10.1186/s12864-024-10312-3 (PMC11046876; doi:10.1186/s12864-024-10312-3)
Supplement: Supplementary file 24 — Supplementary Material 24: Table S22 [file 12864_2024_10312_MOESM24_ESM.pdf]

Table S22: Tukey's post-hoc pairwise contrasts on the significant 3-way interaction (enzyme*supergroup*genomic category). The column contrast indicates the variables being compared with the post-hoc test and the first three columns indicate which factors are fixed (the factor being tested is represented with an asterisk). For each comparison we provide its p-value. Significant p-values are in bold.

Supergroup	Genomic Category	Enzyme	Contrast	t-ratio	p-value
Plants	*	AlfI	Exonic - Intergenic	28.83	**<0.001**
Plants	*	AlfI	Exonic - Intronic	17.50	**<0.001**
Plants	*	AlfI	Intergenic - Intronic	-11.34	**<0.001**
Plants	*	CspCI	Exonic - Intergenic	22.67	**<0.001**
Plants	*	CspCI	Exonic - Intronic	15.65	**<0.001**
Plants	*	CspCI	Intergenic - Intronic	-7.02	**<0.001**
Plants	*	BaeI	Exonic - Intergenic	20.47	**<0.001**
Plants	*	BaeI	Exonic - Intronic	11.94	**<0.001**
Plants	*	BaeI	Intergenic - Intronic	-8.53	**<0.001**
Protostomes	*	AlfI	Exonic - Intergenic	14.70	**<0.001**
Protostomes	*	AlfI	Exonic - Intronic	12.90	**<0.001**
Protostomes	*	AlfI	Intergenic - Intronic	-1.79	0.998
Protostomes	*	CspCI	Exonic - Intergenic	16.75	**<0.001**
Protostomes	*	CspCI	Exonic - Intronic	15.57	**<0.001**
Protostomes	*	CspCI	Intergenic - Intronic	-1.18	1.000
Protostomes	*	BaeI	Exonic - Intergenic	14.24	**<0.001**
Protostomes	*	BaeI	Exonic - Intronic	12.77	**<0.001**
Protostomes	*	BaeI	Intergenic - Intronic	-1.47	1.000
Deuterostomes	*	AlfI	Exonic - Intergenic	19.49	**<0.001**
Deuterostomes	*	AlfI	Exonic - Intronic	13.77	**<0.001**
Deuterostomes	*	AlfI	Intergenic - Intronic	-5.72	**<0.001**
Deuterostomes	*	CspCI	Exonic - Intergenic	19.58	**<0.001**
Deuterostomes	*	CspCI	Exonic - Intronic	14.84	**<0.001**
Deuterostomes	*	CspCI	Intergenic - Intronic	-4.74	**<0.001**
Deuterostomes	*	BaeI	Exonic - Intergenic	20.29	**<0.001**
Deuterostomes	*	BaeI	Exonic - Intronic	14.56	**<0.001**
Deuterostomes	*	BaeI	Intergenic - Intronic	-5.73	**<0.001**
*	Exonic	AlfI	Plants - Protostomes	8.20	**<0.001**
*	Exonic	AlfI	Plants - Deuterostomes	9.29	**<0.001**
*	Exonic	AlfI	Protostomes - Deuterostomes	0.09	1.000
*	Exonic	CspCI	Plants - Protostomes	4.03	**0.009**
*	Exonic	CspCI	Plants - Deuterostomes	5.68	**<0.001**
*	Exonic	CspCI	Protostomes - Deuterostomes	1.23	1.000
*	Exonic	BaeI	Plants - Protostomes	3.96	**0.012**
*	Exonic	BaeI	Plants - Deuterostomes	4.05	**0.009**
*	Exonic	BaeI	Protostomes - Deuterostomes	-0.43	1.000
*	Intergenic	AlfI	Plants - Protostomes	-1.76	0.999
*	Intergenic	AlfI	Plants - Deuterostomes	-2.09	0.962

*	Intergenic	AlfI	Protostomes - Deuterostomes	-0.13	1.000
*	Intergenic	CspCI	Plants - Protostomes	-0.74	1.000
*	Intergenic	CspCI	Plants - Deuterostomes	-1.19	1.000
*	Intergenic	CspCI	Protostomes - Deuterostomes	-0.38	1.000
*	Intergenic	BaeI	Plants - Protostomes	-0.86	1.000
*	Intergenic	BaeI	Plants - Deuterostomes	-0.88	1.000
*	Intergenic	BaeI	Protostomes - Deuterostomes	0.10	1.000
*	Intronic	AlfI	Plants - Protostomes	4.51	**0.002**
*	Intronic	AlfI	Plants - Deuterostomes	3.34	0.094
*	Intronic	AlfI	Protostomes - Deuterostomes	-1.83	0.997
*	Intronic	CspCI	Plants - Protostomes	3.10	0.187
*	Intronic	CspCI	Plants - Deuterostomes	1.58	1.000
*	Intronic	CspCI	Protostomes - Deuterostomes	-2.02	0.979
*	Intronic	BaeI	Plants - Protostomes	3.78	**0.022**
*	Intronic	BaeI	Plants - Deuterostomes	2.51	0.678
*	Intronic	BaeI	Protostomes - Deuterostomes	-1.85	0.997
Plants	Exonic	*	AlfI - CspCI	4.74	**<0.001**
Plants	Exonic	*	AlfI - BaeI	6.82	**<0.001**
Plants	Exonic	*	CspCI - BaeI	2.08	0.957
Protostomes	Exonic	*	AlfI - CspCI	-1.89	0.993
Protostomes	Exonic	*	AlfI - BaeI	0.28	1.000
Protostomes	Exonic	*	CspCI - BaeI	2.16	0.922
Deuterostomes	Exonic	*	AlfI - CspCI	-0.36	1.000
Deuterostomes	Exonic	*	AlfI - BaeI	-0.61	1.000
Deuterostomes	Exonic	*	CspCI - BaeI	-0.26	1.000
Plants	Intergenic	*	AlfI - CspCI	-1.42	1.000
Plants	Intergenic	*	AlfI - BaeI	-1.54	1.000
Plants	Intergenic	*	CspCI - BaeI	-0.12	1.000
Protostomes	Intergenic	*	AlfI - CspCI	0.16	1.000
Protostomes	Intergenic	*	AlfI - BaeI	-0.18	1.000
Protostomes	Intergenic	*	CspCI - BaeI	-0.34	1.000
Deuterostomes	Intergenic	*	AlfI - CspCI	-0.27	1.000
Deuterostomes	Intergenic	*	AlfI - BaeI	0.19	1.000
Deuterostomes	Intergenic	*	CspCI - BaeI	0.46	1.000
Plants	Intronic	*	AlfI - CspCI	2.89	0.273
Plants	Intronic	*	AlfI - BaeI	1.26	1.000
Plants	Intronic	*	CspCI - BaeI	-1.63	1.000
Protostomes	Intronic	*	AlfI - CspCI	0.78	1.000
Protostomes	Intronic	*	AlfI - BaeI	0.15	1.000
Protostomes	Intronic	*	CspCI - BaeI	-0.63	1.000
Deuterostomes	Intronic	*	AlfI - CspCI	0.71	1.000
Deuterostomes	Intronic	*	AlfI - BaeI	0.17	1.000
Deuterostomes	Intronic	*	CspCI - BaeI	-0.53	1.000
