# Supplementary material for: Genome composition and GC content influence loci distribution in reduced representation genomic studies
Source: BMC Genomics. 2024 Apr 25;25:410. doi: 10.1186/s12864-024-10312-3 (PMC11046876; doi:10.1186/s12864-024-10312-3)
Supplement: Supplementary file 25 — Supplementary Material 25: Figure S1 [file 12864_2024_10312_MOESM25_ESM.pdf]

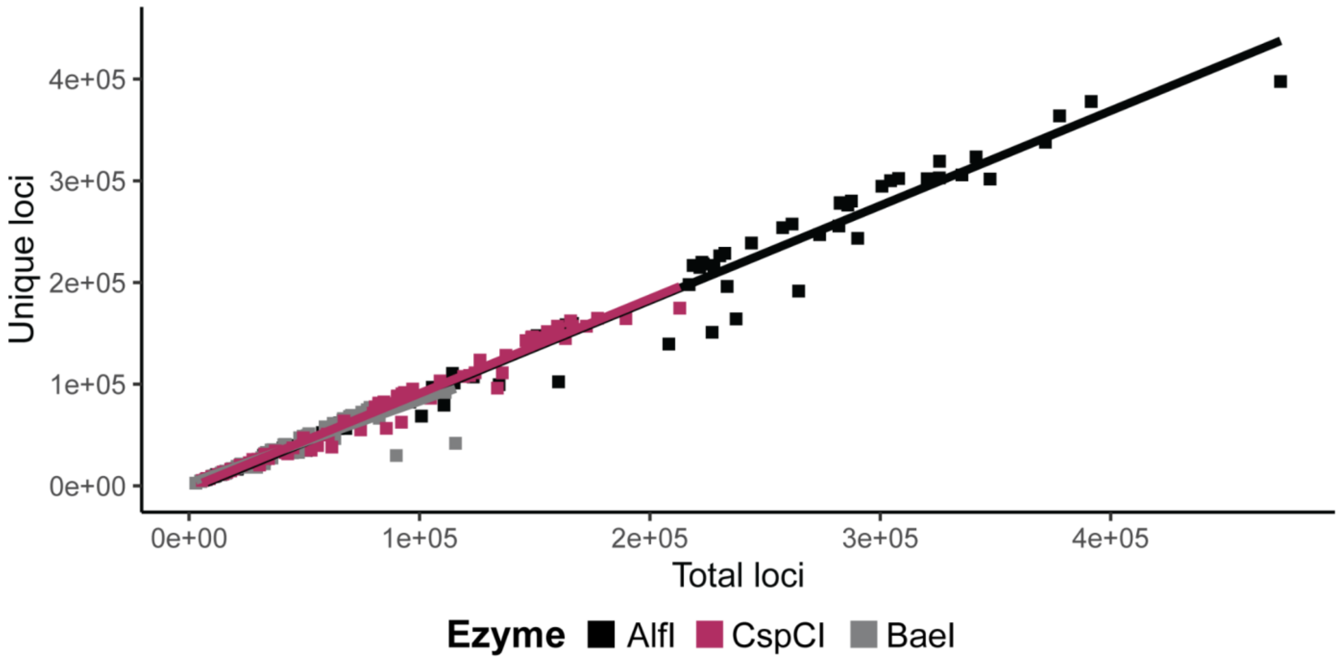

Figure S1: Linear regression between total and unique loci in the 80 analyzed genomes. AlfI (r = 0.99, p <0.001), CspCI (r = 0.99, p <0.001), BaeI (r = 0.92, p <0.001).
