# Supplementary material for: Genome composition and GC content influence loci distribution in reduced representation genomic studies
Source: BMC Genomics. 2024 Apr 25;25:410. doi: 10.1186/s12864-024-10312-3 (PMC11046876; doi:10.1186/s12864-024-10312-3)
Supplement: Supplementary file 26 — Supplementary Material 26: Figure S2 [file 12864_2024_10312_MOESM26_ESM.pdf]

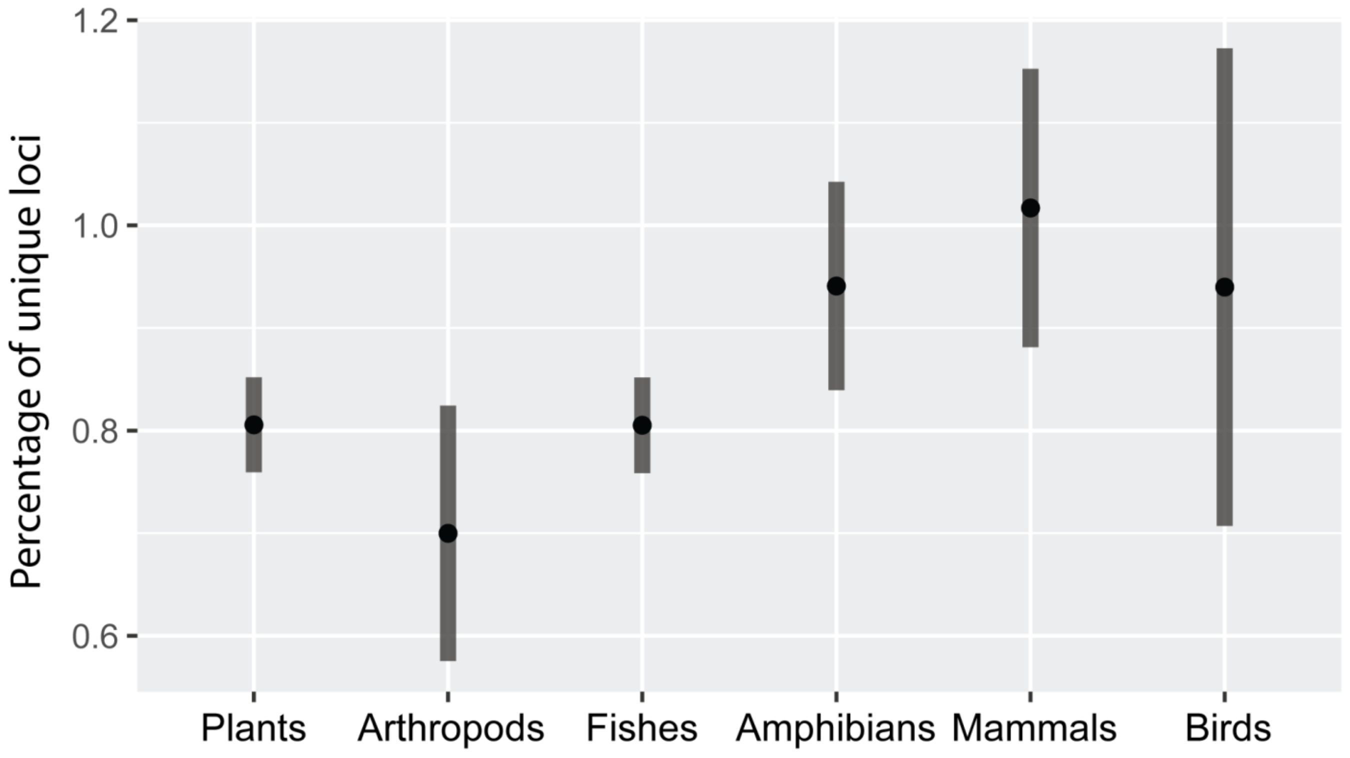

Figure S2: Predicted values of the percentage of unique loci in plants, arthropods, fishes, amphibians, mammals and birds with the group model (Table S8). Mean values are marked with a dot and their 95% confidence intervals are represented with the lines.
