# Supplementary material for: Genome composition and GC content influence loci distribution in reduced representation genomic studies
Source: BMC Genomics. 2024 Apr 25;25:410. doi: 10.1186/s12864-024-10312-3 (PMC11046876; doi:10.1186/s12864-024-10312-3)
Supplement: Supplementary file 27 — Supplementary Material 27: Figure S3 [file 12864_2024_10312_MOESM27_ESM.pdf]

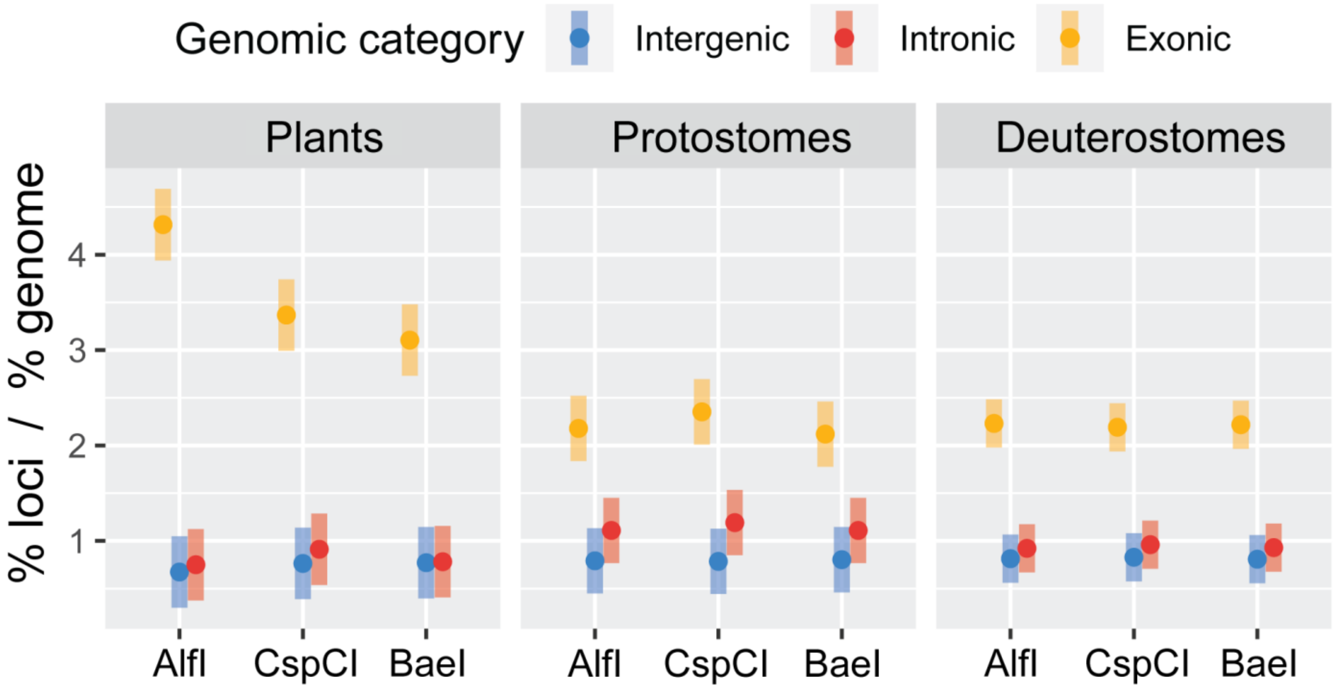

Figure S3: Predicted values of the ratio between the percentage of unique loci in a genomic category and the percentage of genome in the same genomic category with the GLMM provided in Table S13. Mean values are marked with a dot and their 95% confidence intervals are represented with lines.
