# Supplementary material for: Genome composition and GC content influence loci distribution in reduced representation genomic studies
Source: BMC Genomics. 2024 Apr 25;25:410. doi: 10.1186/s12864-024-10312-3 (PMC11046876; doi:10.1186/s12864-024-10312-3)
Supplement: Supplementary file 28 — Supplementary Material 28: Figure S4 [file 12864_2024_10312_MOESM28_ESM.pdf]

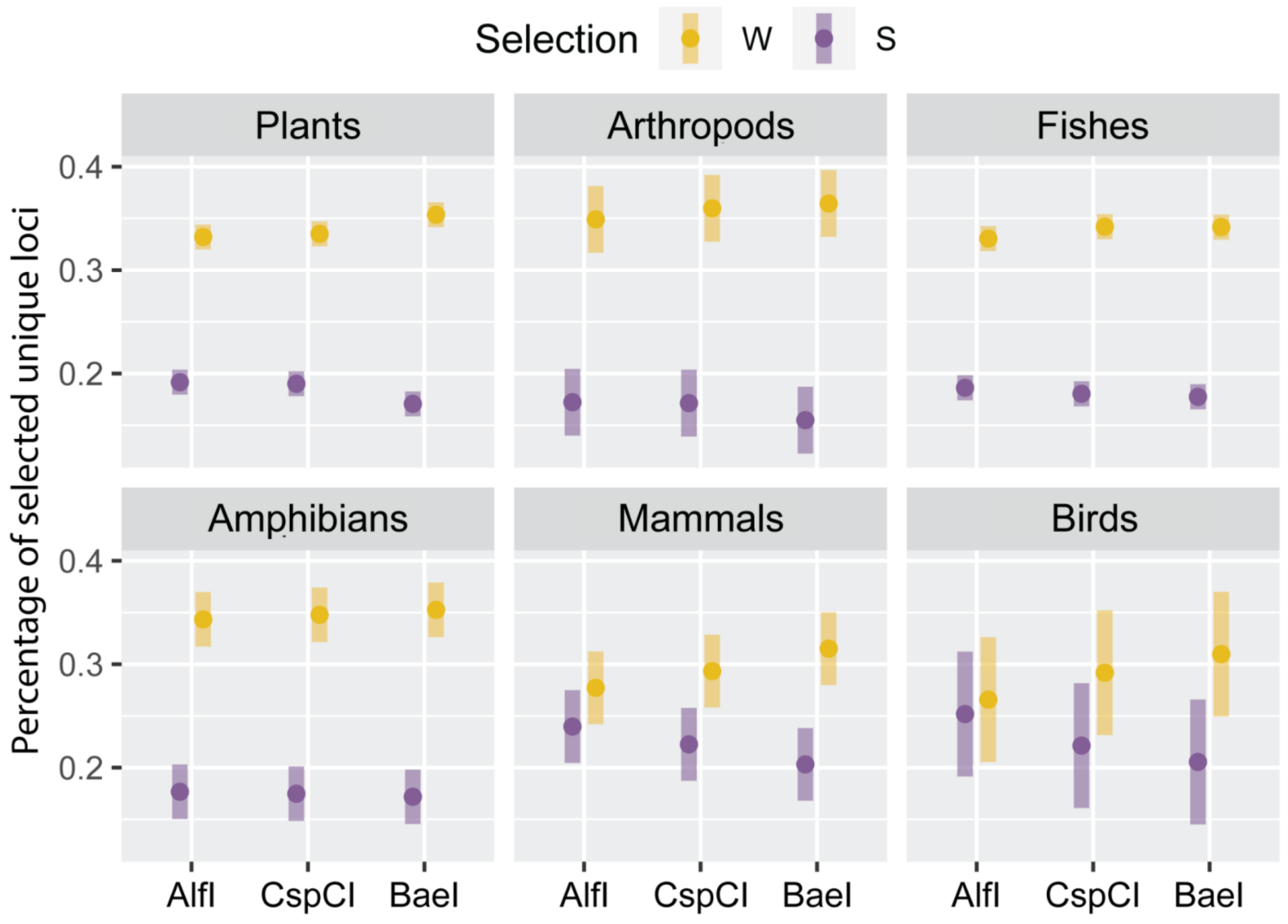

Figure S4: Predicted values of the selected unique loci after secondary reduction with the Group model provided in Table S17. Mean values are marked with a dot and their 95% confidence intervals are represented with lines.
